# Unpacking mathematical gender stereotypes: trends and directions from 25 years of research

**DOI:** 10.3389/fpsyg.2025.1660583

**Published:** 2025-11-18

**Authors:** Özge Nurlu

**Affiliations:** Department of Elementary Education, Faculty of Education, Erzincan Binali Yıldırım University, Education Faculty, Erzincan, Türkiye

**Keywords:** mathematical gender stereotypes, gender bias, stereotype threat, mathematics education, systematic review

## Abstract

Gender norms shape multiple domains, including mathematics—long framed as a male-dominated field—thereby fostering pervasive mathematical gender stereotypes (MGS) that affect individuals’ participation and achievement. This study aims to systematically synthesize empirical research published between 1999 and 2024, indexed in Web of Science, written in English, and available in full text. Only articles explicitly examining MGS were included; studies focused on broader STEM stereotypes, non-English publications, records without full-text access, and papers outside the specified time window were excluded. Limitations include the absence of protocol pre-registration—although inclusion/exclusion criteria and the analysis plan were specified in writing prior to the search and PRISMA 2020 guidelines were followed—and the unavoidable subjectivity in interpretation and categorization despite established inter-coder reliability. Analyses indicate that most studies are situated in psychology, frequently employ experimental designs, and primarily sample university students. Surveys dominate data collection, and parametric inferential statistics are commonly used. Geographically, the literature is concentrated in Western countries—particularly the United States and Germany—with limited contributions from the Global South. Publication counts fluctuate over time, with notable peaks in 2012 and 2022. Conceptually, the literature converges on two principal axes: (i) belief/domain-ownership formulations centered on male superiority and (ii) process-based formulations centered on ST. Less frequently examined yet theoretically informative extensions include endorsement, internalization, counter-stereotypic role models, and stereotype lift. Across qualitative, descriptive, correlational, mediation, meta-analytic, and experimental evidence, findings consistently cluster around these axes, with stereotype endorsement and MGS occupying central positions. Taken together, the results underscore the need for future research that is more interdisciplinary, cross-cultural, and methodologically diverse to more comprehensively address MGS.

## 1 Introduction

Societies often categorize individuals based on particular traits, assigning attributes to social groups that are widely accepted regardless of whether all members of that group actually possess such characteristics (Dökmen, 2017). The concept of “stereotype” was introduced into academic discourse by Lippmann (1922), who defined it as a mental image formed in the minds of individuals—an exaggerated belief or generalization, often based on a single feature of a group or individual (Lippmann, 2009). These stereotypes, which mix elements of truth and distortion, pose serious challenges to discerning the reality of the traits attributed to certain groups (Lippmann, 2009).

Among the most persistent and socially embedded stereotypes are those based on race, religion, and gender. For example, the portrayal of African Americans as lazy and poor reflects an ethnic stereotype (Smith, 1990); viewing Alevis as ill-fated represents a sectarian stereotype (Uyanık, 2012); and associating Muslims with violence or terrorism exemplifies a religious stereotype (Sides and Gross, 2013). Similarly, deeply rooted and persistent societal beliefs contribute to the formation and maintenance of gender-based stereotypes.

Based on characteristics ascribed to women and men, distinctions have emerged across social roles and professions. Women are often described in terms of beauty, grace, and emotionality, and characterized as passive, dependent, and self-sacrificing, whereas men are typically associated with traits like assertiveness, rationality, toughness, and dominance (Külahçı, 1989). These perceptions help reinforce traditional gender roles in both domestic and professional spheres, portraying men as breadwinners and women as caretakers. Accordingly, women are generally linked with communal traits (i.e., social qualities), while men are associated with agency (i.e., autonomy) (Eagly and Steffen, 1984). As a result, certain professions, such as teaching or nursing, are perceived as more appropriate for women, while others, like engineering or law, are more strongly associated with men. Likewise, academic disciplines such as mathematics have traditionally been linked to male identity, contributing to the widespread belief that men possess greater competence and success in mathematics (Beilock et al., 2010).

Historically, the association of mathematics with male identity has reinforced the pervasive stereotype that men are more competent and successful in this domain (Beilock et al., 2010). Given mathematics’ gatekeeping function for socially and economically prestigious careers (Keller and Dauenheimer, 2003; Martinot and Désert, 2007), construing it as a “male” field can undermine girls’ performance on high-stakes transition examinations and, consequently, their educational and occupational choices (Jacobs, 2005). Moreover, mathematical stereotypes operate differentially across intersecting axes of identity—particularly race/ethnicity and gender—shaping both the magnitude and the form of inequality in context-sensitive ways. In this regard, ethnicity may moderate the association between perceptions of academic sexism and academic self-concepts: some girls simultaneously belong to multiple devalued social groups. For example, Latina girls are members of both an ethnic and a gender group linked to negative stereotypes about mathematical competence. This “double-minority” status may heighten sensitivity to both ethnic- and gender-based discrimination, increasing the likelihood of recognizing sexism (Kane, 2000) and amplifying its detrimental effects on academic self-concepts. Consistent with this account, prior research shows that Latina women are more susceptible to gender based stereotype threat (ST) effects than European American women (Gonzales et al., 2002), suggesting that lower ethnic status can increase vulnerability to MGS relative to women from higher-status ethnic groups.

Beyond these socially constructed beliefs, gender has become a critical variable in academic research—particularly in mathematics—where studies have focused on affective, cognitive, and performance-related gender differences. While some research suggests no significant difference in mathematics achievement between male and female students (Hyde et al., 2008), others report differences in specific domains. Studies in cognitive areas such as problem-solving and mathematical reasoning often point to male students having an advantage (Gallagher et al., 2000; Geary et al., 2000). Affective variables—like mathematics anxiety (Barroso et al., 2021), beliefs (Suthar and Tarmizi, 2010), self-efficacy (Skaalvik and Skaalvik, 2006), attitudes (Hwang and Son, 2021) and perceptions about tasks (Bianca and Spagnolo, 2024)—also influence mathematical performance. Findings generally suggest that male students tend to exhibit more favorable affective traits, which positively affect their mathematical outcomes (Markovits and Forgasz, 2017; Miller and Bichsel, 2004; Mozahem et al., 2021; Wilkins and Ma, 2003). Furthermore, several studies have documented gender differences in mathematics performance, often in favor of male students (Lu et al., 2023; Van de Gaer et al., 2008).

Given these patterns, it becomes important to explore the factors underlying such differences. One line of inquiry attributes gender disparities in mathematics to biological distinctions, such as chromosomal or hormonal differences (Berenbaum et al., 2012; Ross et al., 2006). However, the validity of these explanations is contested. Ceci et al. (2009), for example, argue that biological studies on mathematical performance often yield inconsistent and inconclusive results. If gender differences in mathematics were primarily biologically determined, they would likely be consistent across cultures, generations, and educational systems.

Supporting this argument, Else-Quest et al. (2010) conducted a meta-analysis of international data from TIMSS and PISA and found only small average effect sizes for gender differences in mathematics achievement—though these varied widely by country. Their findings suggest that societal factors such as school enrolment equality, female representation in research, and political participation are key predictors of gender disparities in mathematics. Other meta-analyses echo this view, finding that the supposed male superiority in mathematics has diminished over time (Hyde et al., 1990; Hyde, 1981; Lindberg et al., 2010), and that such differences become more pronounced in adolescence, likely due to increased exposure to cultural influences (Fan et al., 1997). Caplan and Caplan (2005) argue that observed gender differences in mathematical ability are shaped more by experience and environment than biology. Cultural transmission plays a significant role in perpetuating stereotypes across generations through media such as television (Hall and Suurtamm, 2020; Wille et al., 2018), children’s books (Ladd, 2011; Nurlu-Üstün and Uzuner-Yurt, 2023), textbooks (Guichot-Reina and De la Torre-Sierra, 2023; Moser and Hannover, 2014; Nurlu, 2021), parental attitudes (Herbert and Stipek, 2005; Tiedemann, 2000; Tomasetto et al., 2015), and teacher interactions (Chionidou-Moskofoglou and Chatzivasiliadou-Lekka, 2008; Heyder et al., 2019; Keller, 2001; Mittelberg et al., 2011; Nurlu-Üstün and Aksoy, 2022).

Although the literature on MGS spans a broad range of topics and approaches, this diversity complicates efforts to assess the field’s current status. Comprehensive syntheses can provide a holistic understanding of the issue, inform educational policy and classroom practice, and raise societal awareness. Moreover, such reviews can guide researchers by mapping the existing literature (Ulutaş and Ubuz, 2008), identifying research gaps, and suggesting new directions (Çiltaş et al., 2012; Suri and Clarke, 2009).

In light of this, the present study aims to systematically examine empirical research on MGS. It specifically seeks to answer the following questions:

1. What is the disciplinary distribution of articles on MGS?
2. What is the thematic focus of these articles?
3. What research methodologies and designs are employed?
4. What are the characteristics of the study samples?
5. a. What data collection instruments are used?
  b. To what extent do these instruments report reliability and validity evidence (e.g., internal consistency, structural validity, test-retest/split-half), and what are the typical values and reporting coverage by instrument family?
6. What types of data analysis methods are applied?
7. What is the geographical distribution of the studies?
8. How has the publication frequency changed over time?
9. What are the definitional axes of “stereotype” in these articles, and how prevalent is each?
10. What types of conclusions regarding gender stereotypes in mathematics are reported across studies?

## 2 Methods

### 2.1 Research design

This study adopts a systematic review methodology to compile and analyze peer-reviewed journal articles that focus on MGS. A systematic review is a rigorous and structured method of synthesizing existing research to answer a clearly defined research question. This process involves identifying, selecting, and critically appraising relevant studies based on predetermined inclusion and exclusion criteria, and follows a transparent and replicable procedure (Higgins and Green, 2011).

### 2.2 Data source

The Web of Science database was selected as the sole data source for this review due to its comprehensive indexing of high-impact scholarly journals, particularly those included in the Social Sciences Citation Index (SSCI) and Emerging Sources Citation Index (ESCI). These indices are widely regarded for their academic credibility and coverage of rigorous, peer-reviewed publications. This approach was intended to enable a focused and reproducible search of peer-reviewed outlets. It should be acknowledged, however, that Scopus, ERIC, and PsycINFO index partially non-overlapping corpora (e.g., education-focused venues, psychology-specific journals, practitioner outlets, and conference proceedings) that may not be fully covered by WoS. The search was last conducted in October 2024. No other databases or sources were used.

All retrieved records were screened for eligibility by the author based on predefined inclusion and exclusion criteria. Screening was performed manually by reviewing titles and abstracts, followed by full-text assessment for potentially eligible studies. As this study is single-authored, no independent dual screening was performed. No automation tools were used in the screening process.

The literature search was conducted using the keywords “MGS” and “sex stereotype math.” The search was finalized in October 2024. A total of 343 articles were retrieved using the first keyword, and 408 articles were identified with the second. After removing 70 duplicates, the remaining records were screened based on predefined criteria.

**FIGURE 1:**
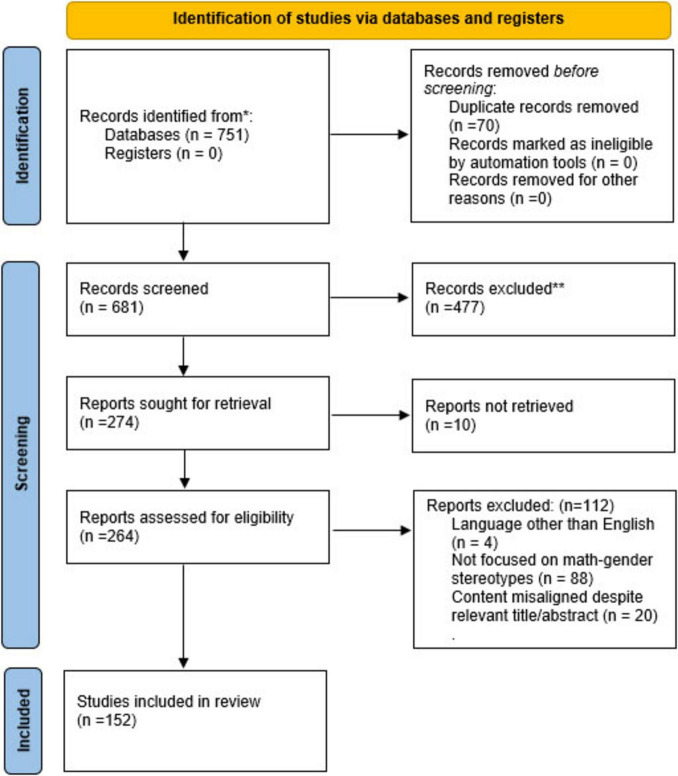
PRISMA flow diagram illustrating the identification, screening, eligibility assessment, and inclusion of articles in the systematic review.

Figure 1 presents the PRISMA flow diagram, which details the flow of information through the phases of the systematic review.

Only studies that included the terms “math and gender stereotypes,” “mathematics and gender stereotypes,” or “mathematical gender stereotypes” in their titles and/or abstracts were retained for further analysis. Following this initial screening, 274 articles were shortlisted for a more detailed eligibility assessment.

The following inclusion criteria were applied to refine the final list of studies:

#### 2.2.1 Full-text accessibility

Articles without full-text access were initially excluded. Authors of these papers were contacted directly. Of the 12 inaccessible studies, only two authors responded and provided the full text. The remaining 10 articles were excluded due to non-availability.

#### 2.2.2 Language

Only studies published in English were included. As a result, four studies written in German, Russian, and Czech were excluded due to language barriers.

#### 2.2.3 Topical relevance

Only studies that explicitly focused on MGS were included. Consequently, 88 articles that discussed gender stereotypes in broader STEM fields were excluded. In addition, although the titles and/or abstracts of 20 studies referred to mathematics and gender stereotypes, these studies were excluded from the analysis as their content was not deemed sufficiently aligned with the theme of MGS.

After applying these inclusion and exclusion criteria, 152 articles remained and were included in the final review. The full list of the analyzed articles is provided in Supplementary Appendix 1.

### 2.3 Data analysis

**FIGURE 2:**
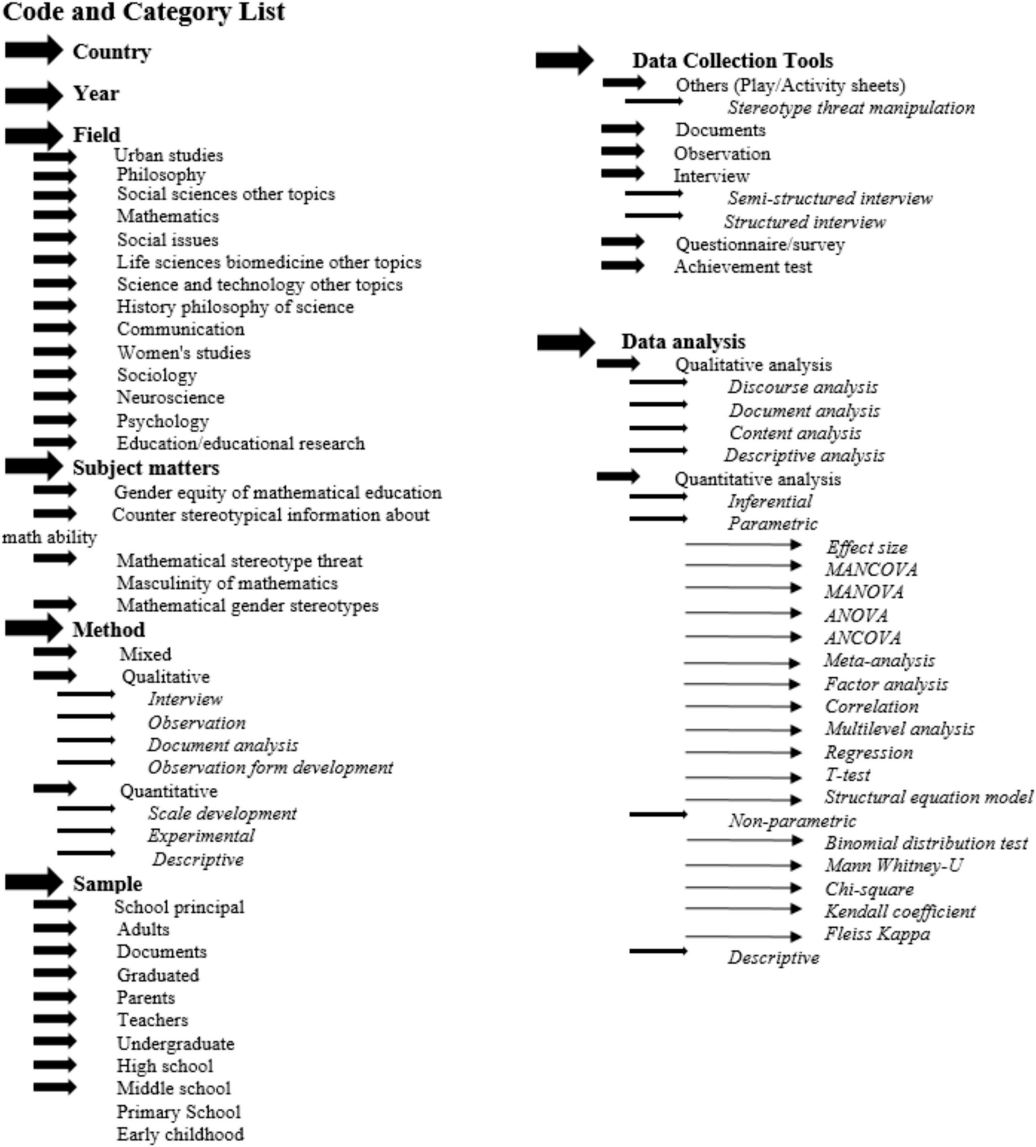
Visual representation of the coding and categorization scheme employed in the systematic review.

Data were extracted independently by one reviewer from each report. The reviewer systematically collected information based on predefined criteria and a coding framework. Although this systematic review was not prospectively registered, the research was guided by the code and category framework developed by Baş and Özturan Sağırlı (2017). No automation tools were used in the data codding process. The code and category list used for analyzing each article is presented in Figure 2.

The framework presented in Figure 2 encompasses eight categories: field, subject matter, methodology, sample, data collection tools, data analysis methods, year, and country. Meta-analysis was not conducted due to the data’s unsuitability for quantitative synthesis.

For each synthesis category (e.g., discipline, methodology, sample, data collection tools, country, year), all included studies were reviewed and coded according to a predefined coding framework. The studies were tabulated and categorized under relevant headings. No studies were excluded from individual syntheses unless they lacked information specific to the category being analyzed.

However, in some studies, MGS were not directly measured using a specific instrument (e.g., questionnaire, scale, or test), but were introduced through experimental manipulation within the research design. In these cases, stereotypes were treated as an independent variable; however, no measurement tool or statistical analysis related to the stereotype variable was reported. Therefore, “ST manipulation” was noted as the data collection tool, while the analysis section was left blank for these studies.

In addition to these structured extractions, we conducted an integrated qualitative synthesis covering stereotype-focused definitions, stereotype-related measurement instruments and their reported reliability, and the substantive findings of each study. All three components were analyzed with the same descriptive-interpretive thematic coding approach. In the first coding cycle, texts were read closely and explicit definitions and conceptual framings of stereotypes, the instruments/scales employed together with their psychometric reports (e.g., internal consistency, structural validity, test-retest), and each study’s principal findings were open-coded. In the second cycle, initial codes were clustered into hierarchical schemes reflecting definitional axes and subthemes, measurement types/instrument families and reported reliability indicators, and direction of findings (female-disadvantaging/female-advantaging/null/mixed) alongside the domain of effect (e.g., performance, attitudes/anxiety, selection/intention, instructional context).

Although no formal risk-of-bias assessment was conducted due to the qualitative content-analysis design, reliability was supported through several procedures: a pilot calibration on a small, randomly selected subset prior to full coding (to clarify inclusion/exclusion criteria, and stabilize thematic categories); independent double coding of approximately 30% of the corpus (*n* = 45) by a second researcher with expertise in education, using pre-specified codes and categories applied to randomly selected studies from the 152 articles; resolution of discrepancies via discussion and consensus with subsequent revisions to the coding scheme as needed; and computation of inter-coder agreement using the Huberman coefficient (Miles and Huberman, 1994), which yielded 83.5%—a level generally considered acceptable in qualitative content analysis. Throughout, a detailed codebook documenting definitions, rules, and representative excerpts was maintained, all updates were logged. In addition, no formal assessment of risk of bias due to missing results (reporting bias) was conducted, as this study employed qualitative content analysis and included all available data from the selected studies.

The coding process was conducted across 10 categories (Supplementary Appendix 4). The main categories were: field, subject matter, methodology, sample, data collection tools, data analysis, country, year, definitions, and conclusions.

## 3 Findings

This section presents the findings of both the individual studies and the synthesized analyses, developed on the basis of the coding framework provided in Figure 2. In addition, it outlines the definitional axes of the concept of “stereotype” identified across the reviewed publications and reports their prevalence. The section also provides a detailed account of the extent to which the instruments employed in these studies include evidence of reliability and validity, such as internal consistency, structural validity, and test-retest or split-half reliability, together with typical values and reporting coverage by instrument family. Finally, it offers a comprehensive synthesis of the patterns of conclusions reached in the literature regarding MGS, encompassing outcomes related to performance, affective factors, intentions, and instructional contexts.

### 3.1 Distribution of MGS-themed articles according to fields

**FIGURE 3:**
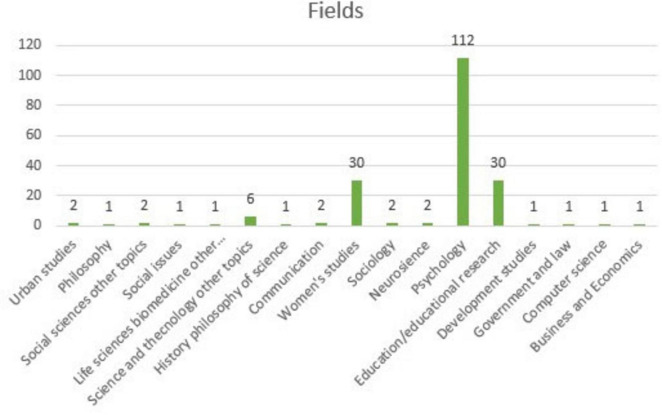
Distribution of the examined articles across research fields.

Figure 3 shows the distribution details of the examined articles across the fields in which they were conducted.

Figure 3 shows that the majority of articles on MGS were published in the field of psychology. Psychology is followed by education/educational sciences and women’s studies. Although there are studies on MGS in various disciplines such as communication or science and technology, the number of published studies appears to be limited.

### 3.2 Distribution of MGS-themed articles according to subject matters

**FIGURE 4:**
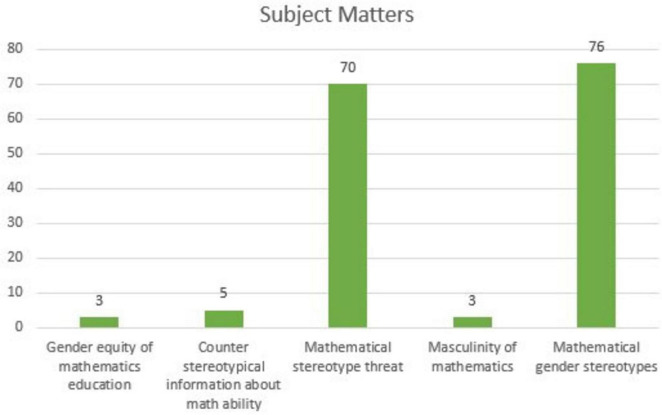
Distribution of subject matters in MGS-themed articles.

Figure 4 shows the findings of the distribution of the subject matters covered in articles on MGS.

As shown in Figure 4, the most frequently addressed subject in MGS-themed articles is MGS themselves. This is followed by topics such as mathematical ST, counter-stereotypical information regarding mathematical ability, the masculinity of mathematics, and gender equity in mathematics education.

### 3.3 Distribution of MGS-themed articles according to research methods/design

**FIGURE 5:**
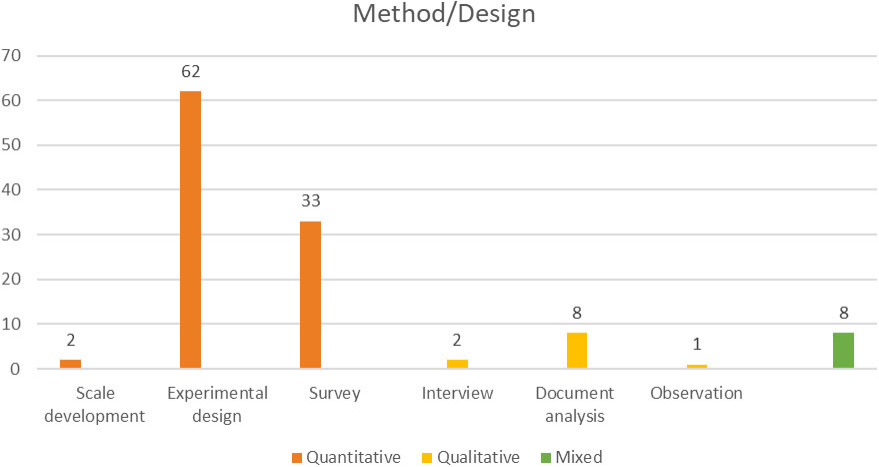
Distribution of research methods/designs in MGS-themed articles.

Figure 5 presents the findings on the distribution of research methods/designs employed in the reviewed articles.

As shown in Figure 5, studies on MGS were predominantly designed as quantitative research. Among quantitative studies, the experimental design was the most commonly used, followed by survey studies and scale development. In contrast, qualitative and mixed-method designs were the least frequently employed research methodologies.

### 3.4 Distribution of MGS-themed articles with respect to the sample

**FIGURE 6:**
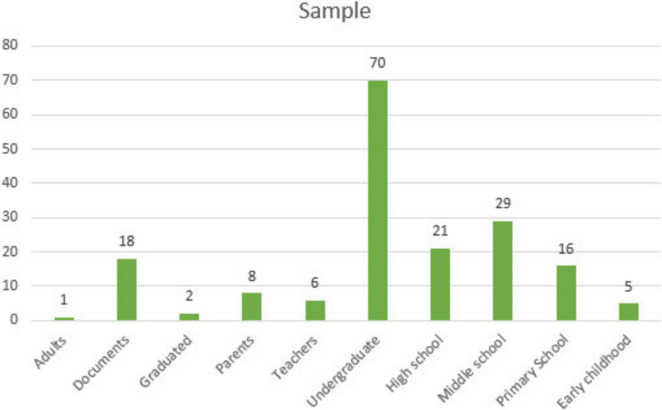
Distribution of samples in MGS-themed articles.

Figure 6 illustrates findings of the distribution of the samples studied in the reviewed articles.

Figure 6 illustrates that the majority of data in MGS-themed articles were collected from undergraduate students. Additionally, some studies focused on high school students, middle school students, primary school students, and documents. However, fewer studies were conducted with adults, graduate students, early childhood students, teachers, and parents.

### 3.5 Distribution of MGS-themed articles with regard to data collection tools

**FIGURE 7:**
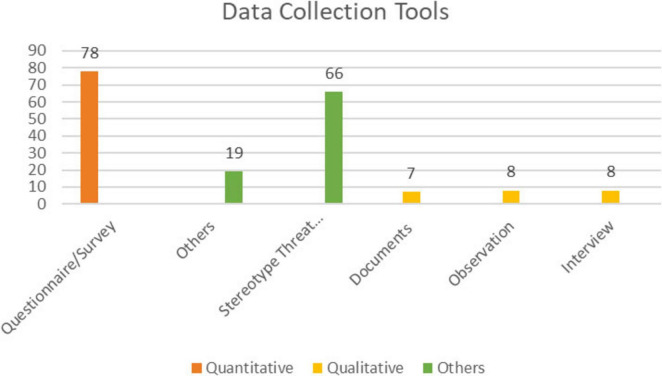
Distribution of data collection tools in MGS-themed articles.

Figure 7 illustrates the distribution of data collection tools used in MGS-themed articles.

As shown in Figure 7, the most commonly used data collection tool in MGS-themed articles was surveys/questionnaires. ST manipulations, categorized under “other,” were also widely utilized. Consistent with the findings on research methods/design, only a few studies employed interviews, observations, and documents.

Table 1 shows the distribution of instrument families and sub-types across the included studies.

**TABLE 1:** Measurement instruments used in studies on mathematical gender stereotypes.

Study number	Title	Author(s)	Year	Family	Name of the measure ment	Reliability	Structural validity	Notes
1	A threat in the classroom: gender stereotype activation and mental-rotation performance in elementary-school children	Sarah Neuburger, Petra Jansen, Martin Heil, Claudia Quaiser-Pohl	2012	Experimental	Stereotype threat manipulation	Not applicable	Not applicable	Instruction Before Post-test: “Boys Better”/“Girls Better”/“No Gender Difference”
2	Stereotype threat effects on Italian girls’ mathematics performance: a failure to replicate	Franca Agnoli, Francesca Melchiorre, Claudio Zandonella Callegher, Gianmarco Altoè	2021	Experimental	Stereotype threat manipulation	Not applicable	Not applicable	Stereotype threat (photographs): 9 male/1 female math icons vs. neutral; count and graph; reminder during post-test.
3	Cognitive, educational and psychological determinants of prospective preschool teachers’ beliefs	Sigrid Blömeke, Simone Dunekacke, Lars Jenßen	2017	Psychometric	Mathematical gender stereotypes	Not reported	CFA: χ^2^(4) = 42.2*, CFI = 0.99, RMSEA = 0.07	6-point Likert
4	Early sources of children’s math achievement in Chile: the role of parental beliefs and feelings about math	M. Francisca del Río, María Inés Susperreguy, Katherine Strasser, Dario Cvencek, Carolina Iturra, Ismael Gallardo, Andrew N. Meltzof	2021	Non-psychometric	Mathematical gender stereotypes (Adult IAT)	Not applicable	Protocol based	
				Non-psychometric	Mathematical gender stereotypes (Explicit)	Not reported	Not reported	
				Non-psychometric	Mathematical gender stereotypes (Child IAT)	Not applicable	Data trim rules were applied	
				Non-psychometric	Mathematical gender stereotypes (pictorial explicit)	Not reported	Not applicable	
5	Masculinity in the public image of physics and mathematics: a new model comparing Japan and England	Yuko Ikkatai, Atsushi Inoue, Azusa Minamizaki, Kei Kano, Euan McKay, Hiromi M. Yokoyama	2021	Non-psychometric	Mathematical gender stereotypes	Not applicable	Not applicable	
6	Urban elementary single-sex math classrooms: mitigating stereotype threat for African American girls	Anica G. Bowe, Christopher D. Desjardins, Lesa M. Covington Clarkson, Frances Lawrenz	2017	Qualitative	Observation	Protocol-guided observations	Not reported	
				Qualitative	Interview	Interviews recorded and transcribed.	Not reported	
7	Students’ gendered perceptions of mathematics in middle grades single-sex and coeducational classrooms	Dennis Kombe, William Bridges, S. Megan Che	2019	Non-psychometric	Mathematical gender stereotypes (WHO and mathematics)	Not applicable	Not applicable	
8	Mathematics self-concept and response pattern in higher education examinations: differences between genders	María Isabel Núñez-Peña, Marta Ramon-Casas, Toni Cunillera, Carlos Campos-Rodríguez	2024	Psychometric	Mathematical gender stereotype endorsement	α = 0.73	Not reported	
9	Self-control capacity moderates the effect of stereotype threat on female university students’ worry during a math performance situation	Alex Bertrams, Christoph Lindner, Francesca Muntoni, Jan Retelsdorf	2022	Experimental	Stereotype threat manipulation	Not applicable	Not applicable	A gender-difference statement (“men consistently outperform women in mathematics”) was embedded in the instructions for female participants.
10	Measuring stereotype threat at math and language arts in secondary school: validation of a questionnaire	Sylwia Bedynska, Piotr Rycielski, Magdalena Jabłonska	2021	Psychometric	Stereotype threat at school scale	Girls α = 0.89 Boys α = 0.88	Girls: χ^2^(11) = 23.93, CFI = 0.99, TLI = 0.98, SRMR = 0.02, RMSEA = 0.04 [90% CI 0.02–0.06]. Boys: χ^2^(12) = 36.86, CFI = 0.98, TLI = 0.96, SRMR = 0.03, RMSEA = 0.06 [90% CI 0.04–0.08].	6-point Likert
11	A study on the influence of the affective domain on the attitudes of middle school students toward mathematics from a gender perspective	M Gutierrez-Aguilar, S Tejeda	2024	Experimental	Stereotype threat manipulation	Not applicable	Not applicable	Videos featuring STEM professionals
				Non-psychometric	Mathematical gender stereotypes	Not applicable	Not applicable	
12	How parents’ stereotypical beliefs relate to students’ motivation and career aspirations in mathematics and language arts	Kathryn Everhart Chaffee, Isabelle Plante	2022	Psychometric	Mathematical gender stereotypes (parents’ ability stereotypes)	α = 0.85 (Math); 0.84 (LA)	Not reported	5-point Likert
13	Divergent effects of system justification salience on the academic self-assessments of men and women	Virginie Bonnot, John T. Jost	2014	Experimental	Stereotype threat manipulation	Not applicable	Not applicable	
				Non-psychometric	Mathematical gender stereotype endorsement	Not applicable	Not applicable	
14	Race, gender, and teacher equity beliefs: construct validation of the attributions of mathematical excellence scale	Erik Jacobson, Dionne Cross Francis, Craig Willey, Kerrie Wilkins-Yel	2022	Psychometric	Mathematical gender stereotypes (attributions of mathematical excellence)	Not reported	Not reported	7-point Likert
15	The effect of the interplay of gender and ethnicity on teachers judgements: does the school subject matter?	Meike Bonefeld, Hannah Kleen, and Sabine Glock	2022	Experimental	Identity cue manipulation via names manipulations	Not applicable	Not applicable	Gender and ethnicity were cued via names (Felix/Hannah = ethnic majority; Murat/Hatice = Turkish minority)
16	Investigating classroom implementation of research-based interventions for reducing stereotype threat in calculus	Elizabeth G. Arnold, Elizabeth A. Burroughs, Jessica M. Deshler	2020	Experimental	Counter-stereotypic role model manipulations	Not applicable	Not applicable	News article about a successful female mathematics student from the same university, with photograph
17	Design and validation of a classroom observation instrument to evaluate the quality of mathematical activity from a gender perspective	Lorena Espinoza Salfate, Gonzalo Guerrero, Joaquim Barbé Farré, Felipe Márquez Salinas	2023	Qualitative	Observation (gender-perspective)	Fleiss’ κ 0.425–0.461 (*p* < 0.001); Kendall’s *W* = 0.489 (*p* < 0.001)	Not reported	
18	Mathematics-gender stereotype endorsement influences mathematics anxiety, self-concept, and performance differently in men and women	Serena Rossi, Iro Xenidou-Dervou, Emine Simsek, Christina Artemenko, Gabriella Daroczy, Hans-Christoph Nuerk, Krzysztof Cipora	2022	Psychometric	Mathematical gender stereotype (mathematics as a male domain subscale)	α = 0.80	Not reported	Endorsement of mathematics as a male domain
19	Stereotype threat may not impact women’s inhibitory control or mathematical performance: providing support for the null hypothesis	Charlotte R. Pennington, Damien Litchfield, Neil McLatchie, Derek Heim	2019	Experimental	Stereotype threat manipulation	Not applicable	Not applicable	Diagnostic framing of gender-linked ability that emphasizes women’s lower performance.
				Experimental	Confrontation of math-gender stereotype manipulation	Not applicable	Not applicable	Women outperform men on visuospatial and mathematical tasks
20	Creating a critical mass eliminates the effects of stereotype threat on women’s mathematical performance	Charlotte R. Pennington, Derek Heim	2016	Experimental	Stereotype threat manipulation	Not applicable	Not applicable	Self-as-target, group-as-target, control
21	Toward gender equality in education—teachers’ beliefs about gender and math	Jana Lindner, Elena Makarova, Deborah Bernhard, Dorothee Brovelli	2022	Psychometric	Mathematical gender stereotypes (teachers’ gender stereotypes toward mathematics)	α = 0.80	Not reported	5-point Likert
22	“Boys press all the buttons and hope it will help”: upper secondary school teachers’ gendered conceptions about students’ mathematical reasoning	Lovisa Sumpter	2016	Non-psychometric	Gendering of reasoning cases	Not applicable	Not applicable	
				Qualitative	Interview	Not reported	Not reported	
23	Reinforcing and reproducing stereotypes? Ethical considerations when doing research on stereotypes and stereotyped reasoning	Mathilde Cecchini	2019	Qualitative	Observation	Not reported	Not reported	
				Qualitative	Document analysis	Not reported	Not reported	
24	Stereotype threat, gender and mathematics attainment: a conceptual replication of Stricker and Ward	Matthew Inglis, Steven O’HaganI	2022	Experimental	Stereotype threat manipulation	Not applicable	Not applicable	Demographic timing: gender-before vs. gender-after
25	Preschoolers’ mathematical play and color preferences: a new window into the development of gendered beliefs about math	Jesús Paz-Albo Prieto, Dario Cvencek, Cristina V. Herranz Llácer, Aránzazu Hervás Escobar, Andrew N. Meltzoff	2017	Qualitative	Observation	Not reported	Not reported	(a) Toy popularity differences between girls and boys; (b) Gender differences in total playtime
				Non-psychometric	Mathematical gender stereotypes	Not applicable	Not applicable	Single-item gendered-belief
26	What does gender has to do with math? Complex questions require complex answers	Luisa Girelli	2023	Review	Review (narrative/ integrative)	Not applicable	Not applicable	Narrative/ integrative review synthesizing evidence on whether mathematics is a gendered domain, the “math male myth,” and sociocultural explanations for the gender gap.
27	K-8 Teachers’ overall and gender-specific beliefs about mathematical aptitude	Yasemin Copur-Gencturk, Ian Thacker, David Quinn	2021	Psychometric	Mathematical gender stereotypes (smart Boys)	α = 0.86	Not reported	
28	The role of perspective-taking in suppressing stereotypes about mathematics	Mana Yamamoto, Takashi Oka	2023	Experimental	Stereotype suppression manipulation	Not applicable	Not applicable	
29	Stereotype threat and gender: math performance in Chinese college students	Ming Tsui, Xiao-ying Xu, Edmond Venator, Yan Wang,	2016	Psychometric	Mathematical gender stereotypes (stereotype-based judgment)	Not reported	Not reported	
				Experimental	Stereotype threat manipulation	Not applicable	Not applicable	
30	Stereotype manipulation effects on math and spatial test performance: a meta-analysis	Randi A. Doyle, Daniel Voyer	2016	Meta-analysis				
31	Gender stereotypes: implicit threat to performance or boost for motivational aspects in primary school?	Johanna Maria Hermann, Regina Vollmeyer	2022	Experimental	Stereotype threat manipulation	Not applicable	Not applicable	Implicit gender cues via gender-specific math word problems
32	Gender stereotypes, performance and identification with math	Irena Smetackova	2015	Psychometric	Mathematical gender stereotypes (gender schemas/beliefs about mathematics)	Not reported	Not reported	
33	Parent-child math anxiety and math-gender stereotypes predict adolescents’ math education outcomes	Bettina J. Casad, Patricia Hale, Faye L. Wachs	2015	Psychometric	Mathematical gender stereotype endorsement	α = 0.80	Not reported	6-point Likert
34	Stereotype threat among girls: differences by gender identity and math education context	Bettina J. Casad, Patricia Hale, Faye L. Wachs	2017	Experimental	Stereotype threat manipulation	Not applicable	Not applicable	
35	The representation of gender stereotypes in Spanish mathematics textbooks for elementary education	Virginia Guichot-Reina, Ana María De la Torre-Sierra	2023	Qualitative	Document Analysis	Not reported	Not reported	
36	Counter-stereotypes and images: an exploratory research and some questions	Christine Morin-Messabel, Se’verine Ferrie‘re, Frederic Martinez, Julie Devif, Laurence Reeb	2017	Experimental	Stereotype threat manipulation	Not applicable	Not applicable	
37	Can gender priming eliminate the effects of stereotype threat? The case of simple dynamic systems	Vivien Lungwitz, Peter Sedlmeier, Marcus Schwarz	2018	Meta-Analysis				
38	Gender representation in the national assessments of mathematical achievements	Monika Grigaliūnienė, Roma Kačinskaitė	2021	Qualitative	Document analysis	Not reported	Not reported	Unit of analysis: contextualized word problems in national mathematics assessments that include gender references.
39	Chronic stereotype threat is associated with mathematical achievement on representative sample of secondary schoolgirls: the role of gender identification, working memory, and intellectual helplessness	Sylwia Bedynska, Izabela Krejtz, Grzegorz Sedek	2018	Psychometric	Chronic stereotype threat	α = 0.89	χ^2^(14) = 73.56, CFI = 0.928, TLI = 0.891, SRMR = 0.047, RMSEA = 0.083 [90% CI 0.065–0.102]	6-point Likert
40	Exploring the nature of teachers’ math-gender stereotypes: the math-gender misconception questionnaire	Anna-Sophia Dersch, Anke Heyder, Alexander Eitel	2022	Psychometric	Mathematical gender stereotypes (math-gender misconceptions questionnaire)	α = 0.82	CFA (3-factor > 1-factor): standardized loadings 0.44–0.88; fit indices: not reported	Each item: agree/disagree × 5-point certainty → misconception score –4; +4; 15 filler “true” items balance acquiescence
41	Implicit math-gender stereotype present in adults but not in 8th grade	Kyle Morrissey, Darcy Hallett, Aishah Bakhtiar, Cheryll Fitzpatrick	2019	Non-psychometric	Mathematical gender stereotypes (explicit)	Not applicable	Not applicable	5-point Likert
				Non-psychometric	Mathematical gender stereotypes (math-language implicit association test)	Not applicable	Not applicable	
42	Can math-gender stereotypes be reduced? A theory-based intervention program with adolescent girls	Fengqing Zhao, Yiyin Zhang, Valeria Alterman, Baoshan Zhang, Guoliang Yu	2018	Experimental	Identity threat model-based intervention	Not applicable	Not applicable	
				Psychometric	Mathematical gender stereotypes	α = 0.80	CFI 0.97; NFI 0.94; GFI 0.92; RMSEA 0.079; Loadings 0.71–0.95	
43	Gender in mathematics: how gender role perception influences mathematical capability in junior high school	Guihua Xie, Xinyu Liu	2023	Non-psychometric	Mathematical gender stereotypes	Not applicable	Not applicable	
44	Determination of primary school teachers’ mathematical gender stereotypes and examination of their reflection on students	Ozge Nurlu Ustun, Naciye Aksoy	2022	Psychometric	Mathematical gender stereotypes (teachers’ gender stereotypes toward mathematics)	Girls form: α = 0.91; boys form: α = 0.88	Not reported	5-point Likert
				Qualitative	Observation (teacher-child dyadic interaction system)	Not reported	Not reported	
				Non-psychometric	Mathematical gender stereotypes (students’ gender stereotype questionnaire)	Not reported	Not reported	
45	Gender-math stereotypes and mathematical performance: the role of attitude toward mathematics and math self-concept	Fang Xie, Yan Yang, Cong Xiao	2023	Psychometric	Mathematical gender stereotype endorsement	α = 0.88	Not reported	7-point Likert
46	Gender differences in young adults’ mathematical performance: examining the contribution of working memory, math anxiety and gender-related stereotypes	Helene Vos, Mila Marinova, Sara C. De L’eon, Delphine Sasanguie, Bert Reynvoet	2023	Psychometric	Mathematical gender stereotypes (implicit association test)	α = 0.89	Protocol Based	
47	Variations of gender-math stereotype content affect women’s vulnerability to stereotype threat	Dustin B. Thoman, Paul H. White, Niwako Yamawaki, Hirofumi Koishi	2008	Experimental	Stereotype threat manipulation	Not applicable	Not applicable	
48	Are parents’ academic gender stereotypes and changes in them related to their perceptions of their child’s mathematical competence?	Hannu Räty, Riitta Kärkkäinen	2011	Non-psychometric	Mathematical gender stereotypes	Not applicable	Not applicable	5-point Likert
49	Mind the gap: framing of women’s success and representation in STEM affects women’s math performance under threat	Emily S. Shaffer, David M. Marx, Radmila Prislin	2013	Psychometric	Stereotype threat-based concerns	α = 0.63	PCA (Varimax): one-factor solution; all item loadings ≥ 0.71; KMO/Bartlett: not reported; CFA: not reported.	7-point Likert
50	Gender differences in persistence and attributions in stereotype relevant contexts	Amy Kiefer, Margaret Shih	2006	Experimental	Stereotype threat manipulation	Not applicable	Not applicable	
51	Effects of salient multiple identities on women’s performance under mathematics stereotype threat	Dana M. Gresky, Laura L. Ten Eyck, Charles G. Lord, Rusty B. McIntyre	2005	Experimental	Stereotype threat manipulation	Not applicable	Not applicable	
52	Understanding the paradox in math-related fields: why do some gender gaps remain while others do not?	Sapna Cheryan	2012	Review	Review (narrative/ integrative)	Not applicable	Not applicable	
53	Culture, context and stereotype threat: a comparative analysis of young Ugandan women in Coed and single-sex schools	Katherine Picho, Jason M. Stephens	2012	Experimental	Stereotype threat manipulation	Not applicable	Not applicable	
54	Influence of item content and stereotype situation on gender differences in mathematical problem solving	Margaret Walsh, Crystal Hickey, Jim Duffy	1999	Experimental	Stereotype threat manipulation	Not applicable	Not applicable	Gender labeling
55	Gender stereotype endorsement and achievement-related outcomes: the role of competence beliefs and task values	Isabelle Plante, Roxane de la Sablonnière, Joshua M. Aronson, Manon Théorêt	2013	Psychometric	Mathematical gender stereotypes (math and language arts)	Math: α (Male) = 0.92, α (Female) = 0.89; Language: α (Male) = 0.91, α (Female) = 0.89	Not reported	7-point Likert
56	The effects of stereotype threat and double-minority status on the test performance of Latino women	Patricia M. Gonzales Hart Blanton Kevin J. Williams	2002	Experimental	Stereotype threat manipulation	Not applicable	Not applicable	Diagnosticity × ST (gender and ethnicity)
57	Reducing stereotype threat in order to facilitate learning	Kathryn L. Boucher, Robert J. Rydell, Katie J. Van Loo, Michael T. Rydell	2012	Experimental	Stereotype threat manipulation	Not applicable	Not applicable	
58	Girls’ performance in mathematics in upper primary schools of Addis Ababa	Tilaye Kassahun, Bedru Kedir	2006	Qualitative	Document analysis	Not reported	Not reported	
59	Parents’ explanations of their child’s performance in mathematics and reading: a replication and extension of Yee and Eccles	Hannu Raty, Johanna Vansk, Kati Kasanen, Riitta Karkkainen	2002	Non-psychometric	Mathematical gender stereotypes	Not applicable	Not applicable	5-point Likert
60	Stereotype threat in the classroom: dejection mediates the disrupting threat effect on women’s math performance	Johannes Keller, Dirk Dauenheimer	2003	Experimental	Stereotype threat manipulation	Not applicable	Not applicable	
61	Stereotype threat among school girls in quasi-ordinary classroom circumstances	Pascal Huguet, Isabelle Re’gner	2007	Experimental	Stereotype threat manipulation	Not applicable	Not applicable	
62	Stereotype internalization, math perceptions, and occupational choices of women with counter-stereotypical university majors	Virginie Bonnot, Jean-Claude Croizet	2007	Non-psychometric	Mathematical Gender Stereotype Endorsement	Not applicable	Not applicable	7-point Likert
				Non-psychometric	Mathematical gender stereotype awareness	Not applicable	Not applicable	7-point Likert
63	Mathematics and gender stereotypes in one Jewish and one Druze Grade 5 classroom in Israel	David Mittelberg, Osnat Rozner, Helen Forgasz	2011	Qualitative	Document analysis	Not reported	Conducted alongside observations; looked for stereotypy indicators	
				Qualitative	Observation	Not reported	Unannounced, randomized visit times to capture typical lessons; multiple sessions; video records; triangulation with interviews and materials	
				Qualitative	Interview	Audio-recorded and transcribed	Protocol standardization (shared opening prompt); audio capture; triangulation with observations	
64	An examination of implicitly activated, explicitly activated, and nullified stereotypes on mathematical performance: it’s not just a woman’s issue	Jessi L. Smith, Paul H. White	2002	Experimental	Stereotype threat manipulation	Not applicable	Not applicable	
65	The negative consequences of threat	Anne C. Krendl, Jennifer A. Richeson, William M. Kelley, Todd F. Heatherton	2008	Experimental	Stereotype threat manipulation	Not applicable	Not applicable	
66	Reducing the impact of stereotype threat on women’s math performance: are two strategies better than one?	Paul R. Jones	2011	Experimental	Stereotype threat manipulation	Not applicable	Not applicable	
67	Discounting the difficult: how high math-identified women respond to stereotype threat	Alexandra C. Lesko, Jennifer Henderlong Corpus	2006	Experimental	Stereotype threat manipulation	Not applicable	Not applicable	
68	French children’s awareness of gender stereotypes about mathematics and reading: when girls improve their reputation in math	Delphine Martinot, Céline Bagès, Michel Désert	2012	Non-psychometric	Indirect role/competence attribution task	Not applicable	Not applicable	Counter balancing; expected age-choice; domain sensitivity expected
				Non-psychometric	Mathematical gender stereotype awareness (direct awareness of others’ beliefs)	Not reported	Not reported	5-point Likert
69	The costs of accepting gender differences: the role of stereotype endorsement in women’s experience in the math domain	Toni Schmader, Michael Johns, Marchelle Barquissau	2004	Experimental	Stereotype threat manipulation	Not applicable	Not applicable	
				Psychometric	Mathematical gender stereotype endorsement	α = 0.88	Not reported	
70	Implicit social cognitions predict sex differences in math engagement and achievement	Brian A. Nosek University of Virginia Frederick L. Smyth	2011	Psychometric	Mathematical gender stereotypes (explicit stereotyping)	α = 0.60	Parallel to implicit stereotyping construct	4-point Likert
				Psychometric	Mathematical gender stereotypes (implicit stereotyping IAT)	Not reported	Not reported	
71	Problems in the pipeline: stereotype threat and women’s achievement in high-level math courses	Catherine Good, Joshua Aronson, Jayne Ann Harder	2008	Experimental	Stereotype threat manipulation	Not applicable	Not applicable	
72	Stereotype threat as validity threat: the anxiety-sex-threat interaction	Ana R. Delgado, Gerardo Prieto	2008	Experimental	Stereotype threat manipulation	Not applicable	Not applicable	
73	Separating implicit gender stereotypes regarding math and language: implicit ability stereotypes are self-serving for boys and men, but not for girls and women	Melanie C. Steffens, Petra Jelenec	2011	Non-psychometric	Mathematical gender stereotypes (GNAT)	Split-half (odd-even go trials): Math *r* = 0.42/0.39; language *r* = 0.34/0.43. α: not applicable	Not applicable	Standardized effect size computation: the difference in mean reaction times between the two critical tasks divided by the overall standard deviation of response-latencies; error trial reaction times are included in the calculation; distractor stimuli from a superordinate category (school) are employed to increase task demands
				Non-psychometric	Mathematical gender stereotype endorsement (explicit math)	Not reported	Not reported	Difference scores rescaled to 1–5; awareness separate
74	Stereotype internalization and women’s math performance: the role of interference in working memory	Virginie Bonnot, Jean-Claude Croizet	2007	Psychometric	Mathematical gender stereotype awareness	Not reported	Not reported	7-point Likert
				Psychometric	Mathematical gender stereotype endorsement	Not reported	Not reported	7-point Likert
75	Women are bad at math, but I’m not, am I?’ Fragile mathematical self-concept predicts vulnerability to a stereotype threat effect on mathematical performance	Friederike X. R. Gerstenberg, Roland Imhoff, Manfred Schmitt	2012	Experimental	Stereotype threat manipulation	Not applicable	Not applicable	
76	Self-affirmation in occupational training: effects on the math performance of French women nurses under stereotype threat	Anne Taillandier-Schmitt, Catherine Esnard, René Mokounkolo	2012	Experimental	Stereotype threat manipulation	Not applicable	Not applicable	
77	Stereotype threat and women’s math performance	Steven J. Spencer, Claude M. Steele, Diane M. Quinn	1999	Experimental	Stereotype threat manipulation	Not applicable	Not applicable	
78	Blatant stereotype threat and women’s math performance: self-handicapping as a strategic means to cope with obtrusive negative performance expectations	Johannes Keller	2002	Experimental	Stereotype threat manipulation	Not applicable	Not applicable	
79	The interplay among stereotypes, performance-avoidance goals, and women’s math performance expectations	Jessi L. Smith	2006	Experimental	Stereotype threat manipulation	Not applicable	Not applicable	
80	Stereotype internalization, math perceptions, and occupational choices of women with counter-stereotypical university majors	Virginie Bonnot, Jean-Claude Croizet	2007	Non-psychometric	Mathematical gender stereotype endorsement	Not applicable	Convergent/ criterion validity	7-point Likert
				Non-psychometric	Mathematical gender stereotype awareness	Not applicable	Face/content validity	7-point Likert
81	Math-gender stereotypes in elementary school children	Dario Cvencek, Andrew N. Meltzoff, Anthony G. Greenwald	2011	Non-psychometric	Mathematical gender stereotypes	α = 0.03	Not reported	
				Psychometric	Mathematical gender stereotypes (Child IAT)	α = 0.74	Age-appropriate group differences (boys show stronger boy-math association); converges with explicit indices and predicts emerging math self-concept patterns.	
82	Stereotype threat reduces motivation to improve: effects of stereotype threat and feedback on women’s intentions to improve mathematical ability	Vincent J. Fogliati, Kay Bussey	2013	Experimental	Stereotype threat manipulation	Not applicable	Not applicable	
83	New directions for research on the role of parents and teachers in the development of gender-related math attitudes: response to commentaries	Elizabeth A. Gunderson, Gerardo Ramirez, Susan C. Levine, Sian L. Beilock	2012	Review	Review (narrative/ integrative)			
84	The effect of negative performance stereotypes on learning	Robert J. Rydell, Michael T. Rydell, Kathryn L. Boucher	2010	Experimental	Stereotype threat manipulation	Not applicable	Not applicable	
85	Revealing stereotype threat effects and women’s maths performance the moderating role of mathematical anxiety	Daniel Pérez-Garín, Antonio Bustillos, Fernando Molero	2017	Experimental	Stereotype threat manipulation	Not applicable	Not applicable	
86	Do parents’ academic gender stereotypes influence whether they intrude on their children’s homework?	Ruchi Bhanot, Jasna Jovanovic	2005	Psychometric	Mathematical gender stereotypes	Mothers: α = 0.85 (mathematics), α = 0.86 (English); Fathers: α = 0.82 (Mathematics), α = 0.78 (English)	Convergent/ criterion validity	5-point Likert
87	Forewarning and forearming stereotype-threatened students	Matthew S. McGlone, Joshua Aronson	2001	Experimental	Stereotype threat manipulation	Not applicable	Not applicable	
88	A beautiful myth? The gendering of being/doing “good at maths”	Heather Mendick	2005	Qualitative	Observation	Not reported	Not reported	
				Qualitative	Interview	Not reported	Not reported	
89	Gender and mathematics: recent development from a Swedish perspective	Gerd Brandell, Gilah Leder, Peter Nyström	2007	Psychometric	Mathematical gender stereotypes (who and mathematics)	Not reported	Careful translation to Swedish; Large, stratified school sample (34 classes in 17 schools; *N* = 747, year 9); administered under standardized conditions	
				Psychometric	Mathematical gender stereotypes (mathematics as a gendered domain)	Not reported	Not reported	
				Qualitative	Interview	Not reported	Verbatim transcription; thematic analysis; conducted by multiple trained researchers; iterative coding	
90	Images of mathematicians: a new perspective on the shortage of women in mathematical careers	Katrina Piatek-Jimenez	2008	Qualitative	Interview	Interviews were audio-recorded and transcribed; coding conducted by the researcher using an inductive, grounded-theory approach	Prolonged engagement/ rapport: researcher was on the teaching team and knew students; coercion mitigation: no influence on formal course assessment; verbatim transcription; pseudonyms used	
91	Shaping stereotypical behavior through the discussion of social stereotypes	Laura G. E. Smith, Tom Postmes	2011	Experimental	Stereotype threat manipulation	Not applicable	Not applicable	
92	Psychological processes underlying stereotype threat and standardized math test performance	Katherine E. Ryan, Allison M. Ryan	2005	Review	Review (narrative/ integrative)			
93	Identity bifurcation in response to stereotype threat: women and mathematics	Emily Pronin, Claude M. Steele, Lee Ross	2004	Experimental	Stereotype threat manipulation	Not applicable	Not applicable	
94	The role of performance-avoidance goals and worry in mediating the relationship between stereotype threat and performance	Amanda B. Brodish, Patricia G. Devine	2009	Experimental	Stereotype threat manipulation	Not applicable	Not applicable	
95	Gender, stereotype threat, and anxiety: psychophysiological and cognitive evidence	Jason W. Osborne	2006	Experimental	Stereotype threat manipulation	Not applicable	Not applicable	
96	Social identity versus reference frame comparisons: the moderating role of stereotype endorsement	Hart Blanton, Charlene Christie, Maureen Dye	2002	Experimental	Stereotype threat manipulation	Not applicable	Not applicable	High salience: gender-difference framing (“women may do worse”); low alliance: neutral standardization.
				Non-psychometric	Stereotype personalizing	*r* = 0.68 (inter-item); α not reported	Not reported	Two items averaged; labels at 0/3/6/9
				Non-psychometric	Stereotype offense	*r* = 0.70 (inter-item); α not reported	Not reported	Two items averaged; labels at 0/3/6/9
				Non-psychometric	Mathematical gender stereotype endorsement math-spatial stereotype endorsement)	*r* = 0.51 (inter-item); α: not reported	Not reported	Items averaged to composite; labels at 0/3/6/9
97	Lazy, dumb, or industrious: when stereotypes convey attribution information in the classroom	Christine Reyna	2000	Review	Review (narrative/ integrative)			
98	An examination of stereotype threat effects on girls’ mathematics performance	Colleen M. Ganley, Leigh A. Mingle, Allison M. Katherine Ryan, Marina Vasilyeva, Michelle Perry	2013	Experimental	Stereotype threat manipulation	Not applicable	Not applicable	
99	The stereotyped task engagement process: the role of interest and achievement motivation	Jessi L. Smith, Carol Sansone, Paul H. White	2007	Experimental	Stereotype threat manipulation	Not applicable	Not applicable	
100	Consuming images: how television commercials that elicit stereotype threat can restrain women academically and professionally	Paul G. Davies. Steven J. Spencer, Diane M. Quinn, Rebecca Gerhardstein	2002	Experimental	Stereotype threat manipulation	Not applicable	Not applicable	
				Non-psychometric	Stereotype activation	Not applicable	Not applicable	
101	Making gender matter: the role of gender-based expectancies and gender identification on women’s and men’s math performance in Sweden	Kimmo Eriksson, Torun Lindholm	2007	Experimental	Stereotype threat manipulation	Not applicable	Not applicable	
				Psychometric	Mathematical gender stereotype endorsement	α = 0.65	Not reported	5-point Likert
102	Confronting math stereotypes in the classroom: its effect on female college students’ sexism and perceptions of confronters	Guy A. Boysen	2013	Experimental	Confrontation of sexist math stereotype manipulation	Not applicable	Not applicable	
103	Can stereotype threat explain the gender gap in mathematics performance and achievement?	Gijsbert Stoet, David C. Geary	2012	Review	Review (narrative/ integrative)			
				Meta-Analysis				
104	The interference of stereotype threat with women’s generation of mathematical problem-solving strategies	Diane M. Quinn, Steven J. Spencer	2001	Experimental	Stereotype threat manipulation	Not applicable	Not applicable	
105	A Q-methodological study of women’s subjective perspectives on mathematics	Debra L. Oswald, Richard D. Harvey	2003	Qualitative	Interview	Not reported	Audio-recorded and transcribed verbatim	
106	A particular resiliency to threatening environments	Michael Inzlicht, Joshua Aronson, Catherine Good, Linda McKay	2006	Experimental	Stereotype threat manipulation	Not applicable	Not applicable	
107	A threatening intellectual environment: why females are susceptible to experiencing problem-solving deficits in the presence of males	Michael Inzlicht, Talia Ben-Zeev	2000	Experimental	Stereotype threat manipulation	Not applicable	Not applicable	
108	Effects of role models from films on short-term ratings of intent, interest, and self-assessment of ability by high school youth: a study of gender-stereotyped academic subjects’	Albert Ziegler, Heidrun Stoeger	2008	Experimental	Stereotype threat manipulation	Not applicable	Not applicable	
109	Do high-achieving female students underperform in private? The implications of threatening environments on intellectual processing	Michael Inzlicht, Talia Ben-Zeev	2003	Experimental	Stereotype threat manipulation	Not applicable	Not applicable	
110	Preschool children’s beliefs about gender differences in academic skills	M. Francisca del Río, Katherine Strasser	2013	Psychometric	Mathematical gender stereotypes	Male α = 0.879, Female α = 0.788	Not reported	
111	Stereotype susceptibility in children: effects of identity activation on quantitative performance	Nalini Ambady, Margaret Shih, Amy Kim, Todd L. Pittinsky	2001	Non-psychometric	Mathematical gender stereotype awareness (implicit)	Not reported	Face/content validity	
				Non-psychometric	Mathematical gender stereotype awareness (explicit)	Not reported	Face/content validity	
112	Stereotype threat and gender differences in performance on a novel visuospatial task	Susan Miller Campbell, Marcia L. Collaer	2009	Experimental	Stereotype threat manipulation	Not applicable	Not applicable	
113	The gendered nature of competence: specific and general aspects of self-knowledge in social contexts’	Laurel J. Bornholt	2000	Comparative	Natural group comparison (coeducational vs. single-sex schools)	Not applicable	Not applicable	
114	Latina and European American girls’ experiences with academic sexism and their self-concepts in mathematics and science during adolescence	Christia Spears Brown, Campbell Leaper	2010	Psychometric	Mathematical gender stereotypes (perceptions of academic sexism)	α = 0.89	Not reported	4-point Likert
115	Stereotype threat and group differences in test performance: a question of measurement invariance	Jelte M. Wicherts, Conor V. Dolan, David J. Hessen	2005	Psychometric Model				
116	Development of children’s math attitudes: gender differences, key socializers, and intervention approaches	Susan C. Levine, Nancy Pantoja	2021	Review	Review (narrative/ integrative)			
117	Does Stereotype Threat Influence performance of girls in stereotyped domains? A meta-analysis	Paulette C. Flore, Jelte M. Wicherts	2015	Meta-Analysis				
118	Effects of gender stereotypes and stereotype threat on children’s performance on a spatial task	Christine K. Shenouda, Judith H. Danovitch	2014	Experimental	Stereotype threat manipulation	Not applicable	Not applicable	
				Non-psychometric	Implicit stereotype story-recall	Not reported	Not reported	
119	Gender role orientation moderates effects of stereotype activation on test performances	Tobias Tempel, Roland Neumann	2015	Experimental	Stereotype threat manipulation	Not applicable	Not applicable	
120	Gender stereotypes about math anxiety: ability and emotional components	M. Jos’e Justicia-Galiano, M. Eva Martín-Puga, Rocío Linares, Santiago Pelegrina	2023	Non-psychometric	Mathematical gender stereotype awareness	Not applicable	Related but distinct from endorsement (*r*≈0.26); serves as “perceived norm” indicator	7-point Likert
				Non-psychometric	Mathematical gender stereotype endorsement	Not applicable	Convergent: with multi-item ability stereotype (*r*≈0.41); discriminant from awareness	7-point Likert
				Psychometric	Mathematical gender stereotypes (explicit)	Not reported	Convergent: correlates with other ability-stereotype indicators; used in moderation tests	5-point Likert
				Psychometric	Mathematical anxiety gender stereotype	α = 0.70 (boys scale), α = 0.72 (girls scale)	Convergent: correlates with general anxiety and math-anxiety measures (*r*≈0.30). Discriminant: distinct from ability-stereotype measures	5-point Likert
121	Gender stereotypes can explain the gender-equality paradox	Thomas Bredaa, Elyès Jouinia, Clotilde Nappc, Georgia Thebaulta	2020	Qualitative	Document analysis	Not reported	Not reported	
122	Gender stereotypes embedded in natural language are stronger in more economically developed and individualistic countries	Clotilde Napp	2023	Qualitative	Document analysis	Not reported	Not reported	
123	Gender stereotypes: implicit threat to performance or boost for motivational aspects in primary school?	Johanna Maria Hermann, Regina Vollmeyer	2022	Experimental	Stereotype threat manipulation	Not applicable	Not applicable	
124	Gendered beliefs about mathematics ability transmit across generations through children’s peers	Alex Eble, Feng Hu	2022	Non-psychometric	Mathematical gender stereotypes (parents’ gender stereotype)	Not applicable	Not applicable	
				Non-psychometric	Mathematical gender stereotypes (child gender stereotype)	Not applicable	Not applicable	
125	Implicit self-stereotyping under eye gaze: the effects of gaze cues on implicit math identity among women	Yusuke Karouji, Takashi Kusumi	2015	Psychometric	Mathematical gender stereotypes (implicit)	Not reported	Not reported	
126	Implicit gender stereotypes and essentialist beliefs predict preservice teachers’ tracking recommendations	Miriam Nürnberger, Josef Nerb, Florian Schmitz, Johannes Keller, Stefan Sütterlin	2016	Psychometric	Mathematical gender stereotypes (explicit)	Not reported	Not reported	6-point Likert
				Psychometric	Mathematical gender stereotypes (implicit)	Not reported	Not reported	
127	Implicit math-gender stereotype present in adults but not in 8th grade	Kyle Morrissey, Darcy Hallett, Aishah Bakhtiar, Cheryll Fitzpatrick	2019	Non-psychometric	Mathematical gender stereotypes (explicit)	Not applicable	Not applicable	5-point Likert
128	Interaction of task difficulty and gender stereotype threat with a spatial orientation task in a virtual nested environment	Craig Allison, Edward S. Redhead, Wai Chan	2017	Experimental	Stereotype threat manipulation	Not applicable	Not applicable	
129	Is Emma or Liam the top scorer in math? The effects of a counter-stereotypical role model on math achievement	Nadia Leroy, Sylvain Max, Pascal Pansu	2022	Non-psychometric	Counter-stereotypic role model manipulations	Not applicable	Not applicable	
130	Leaderboards in a virtual classroom: a test of stereotype threat and social comparison explanations for women’s math performance	Katheryn R. Christy, Jesse Fox	2014	Experimental	Stereotype threat manipulation	Not applicable	Not applicable	
131	Math question type and stereotype threat: evidence from educational settings	Lucy C. Davies, Mark Conner, Constantine Sedikides, Russell R. C	2016	Experimental	Stereotype threat manipulation	Not applicable	Not applicable	
132	Math-gender stereotypes and math-related beliefs in childhood and early adolescence	Maria Chiara Passolunghi, Tania Irene Rueda Ferreira, Carlo Tomasetto	2014	Psychometric	Mathematical gender stereotypes (paper-and-pencil IAT)	Not reported	Protocol Based	
				Psychometric	Mathematical gender stereotypes (explicit math-gender stereotypes)	α = 0.82	Not reported	
133	Mental rotation and mathematics: gender-stereotyped beliefs and relationships in primary school children	Angelica Moè	2018	Non-psychometric	Mathematical gender stereotypes	Not reported	Not reported	
134	Not the sum of its parts: decomposing implicit academic stereotypes to understand sense of fit in math and English	Patricia N. Gilbert, Laurie T. O’Brien, Donna M. Garcia, David M. Marx	2015	Non-psychometric	Mathematical gender stereotypes (GNAT implicit)	Not reported	Not applicable	
135	Numbers for boys and words for girls? Academic gender stereotypes among Chinese parents	Jing Li, Eman Faisal, Ahmed Al Hariri	2022	Psychometric	Mathematical gender stereotypes (stereotype questionnaire)	Not reported	Fathers: χ^2^(48) = 63.34, CFI = 0.990, TLI = 0.986, SRMR = 0.031, RMSEA = 0.030 [90% CI 0.000–0.048] Mothers: χ^2^(48) = 54.11, CFI = 0.996, TLI = 0.993, SRMR = 0.026, RMSEA = 0.018 [90% CI 0.000–0.040]	7-point Likert
136	Parents’ math gender stereotypes and their correlates: an examination of the similarities and differences over the past 25 years	Christine R. Starr, Yannan Gao, Glona Lee, Nayssan Safavian, Charlott Rubach, Anna-Lena Dicke, Jacquelynne S. Ecclesi Sandra D. Simpkins	2022	Non-psychometric	Mathematical gender stereotypes	Not applicable	Not applicable	
137	Rethinking employment discrimination harms	Jessica L. Roberts	2016	Review	Review (narrative/ integrative)	Not applicable	Not applicable	
138	Self-concept explains gender differences in mental rotation performance after stereotype activation	Martina Rahe, Linda Schürmann, Petra Jansen	2023	Psychometric	Mathematical gender stereotypes	α = 0.95	Not reported	
				Experimental	Stereotype threat manipulation	Not applicable	Not applicable	
139	Teacher gender, student gender, and primary school achievement: evidence from 10 francophone African countries	Jieun Lee, Dong-Eun Rhee, Robert Rudolf	2019	Non-psychometric	Mathematical gender stereotypes	Not applicable	Not applicable	
140	The effects of gender composition on women’s experience in math work groups	Sarah S. Grover, Tiffany A. Ito, Bernadette Park	2017	Experimental	Stereotype threat manipulation	Not applicable	Not applicable	Group composition
141	The gender gap in STEM fields: the impact of the gender stereotype of math and science on secondary students’ career aspirations	Elena Makarova, Belinda Aeschlimann, Walter Herzog	2019	Psychometric	Masculinity index	Not reported	Prior validations: Osgood 1957; Hofstätter 1973; Switzerland: Herzog 1998; Makarova & Herzog 2015	7-point Likert
142	The impact of math-gender stereotypes on students’ academic performance: evidence from China	Yilei Luo, Xinqi Chen	2024	Non-psychometric	Mathematical gender stereotypes (self math-gender stereotype)	Not applicable	National, school-representative survey; standard CEPS fielding.	
				Non-psychometric	Mathematical gender stereotypes (perceived parental stereotype)	Not applicable	National, school-representative survey; standard CEPS fielding.	
				Non-psychometric	Mathematical gender stereotypes (perceived societal stereotype)	Not applicable	National, school-representative survey; standard CEPS fielding.	
143	The roots of stereotype threat: when automatic associations disrupt girls’ math performance	Silvia Galdi, Mara Cadinu, Carlo Tomasetto	2014	Experimental	Stereotype threat manipulation	Not applicable	Not applicable	
				Psychometric	Mathematical gender stereotype endorsement (explicit)	Not reported	Not reported	Picture choice
				Psychometric	Mathematical gender stereotype endorsement (implicit)	Not reported	Not reported	
144	The effect of gender stereotypes on young girls’ intuitive number sense	Antonya Marie GonzalezI, Darko Odic, Toni Schmader, Katharina Block, Andrew Scott Baron	2021	Experimental	Stereotype threat manipulation	Not applicable	Not applicable	
				Psychometric	Mathematical gender stereotypes	Not reported	Not reported	Child-friendly pictorial format
145	The effect of mindfulness and stereotype threat in mental rotation: a pupillometry study	Robert Bauer, Leonardo Jost, Petra Jansen	2021	Experimental	Stereotype threat manipulation	Not applicable	Not applicable	
146	The interest gap: how gender stereotype endorsement about abilities predicts differences in academic interests	Isabelle Plante, Paul A. O’Keefe, Joshua Aronson, Catherine Fréchette-Simard, Mélissa Goulet	2019	Psychometric	Mathematical gender stereotypes (gender ability stereotypes)	Math-male domain α = 0.88; language-male domain α = 0.85; math-female domainα = 0.82; language-female domain α = 0.82	Not reported	7-point Likert
147	The negative effects of stereotype threat on women’s spatial ability: the moderating role of resilience	Zhen Wang, Li Zhao, Yiwen Shan, Jian Guan	2024	Experimental	Stereotype threat manipulation	Not applicable	Not applicable	
149	The psychosocial experience of high school girls highly susceptible to stereotype threat: a phenomenological study	Katherine Picho	2016	Qualitative	Observation		Triangulation	
				Qualitative	Interview	Not reported	Transcription; phenomeno logical reduction; code-theme development; contextual/ composite descriptions	
150	The role of implicit gender spatial stereotyping in mental rotation performance	Francesca Guizzoa, Angelica Moèb, Mara Cadinua, Chiara Bertollia	2019	Experimental	Stereotype threat manipulation	Not applicable	Not applicable	
				Non-psychometric	Mathematical gender stereotypes (explicit gender spatial stereotype)	Not applicable	Not applicable	
				Psychometric	Mathematical gender stereotypes (implicit gender spatial stereotyping)	α = 0.72	Not reported	
151	When do gender stereotypes impair math performance? A study of stereotype threat among Ugandan adolescents	Katherine Picho, Toni Schmader	2018	Experimental	Stereotype threat manipulation	Not applicable	Not applicable	
				Psychometric	Mathematical gender stereotype endorsement	Not reported	Not reported	7-point Likert
152	Our future scientists: a review of stereotype threat in girls from early elementary school to middle school	Isabelle Régner, Jennifer R. Steele, Nalini Ambady, Catherine Thinus-Blanc, Pascal Huguet	2014	Review	Review (narrative/ integrative)	Not applicable	Not applicable	

Questionnaire-based approaches clearly dominate: non-psychometric survey items and validated psychometric scales together constitute the largest share. Within the experimental family, stereotype-threat manipulations are the modal sub-type, while other experimental tasks are comparatively rare. Qualitative instruments are infrequently used and, when present, typically serve as supplements rather than primary measures. Taken together, Figure 7 and Table 1 indicate a literature anchored in survey and experimental paradigms with limited qualitative triangulation.

Across the 152 studies, we identified 42 distinct psychometric instruments. Reliability evidence was reported for 23/42 (54.8%)—most often Cronbach’s α—while 19/42 (45.2%) reported none; where provided, evidence was largely confined to internal-consistency coefficients, pointing to a shortfall in psychometric reporting transparency. Qualitative techniques appeared in 23/152 (≈15.1%) studies; 18/23 (≈78.3%) reported no study-specific trustworthiness indicators. The remaining 5/23 (≈21.7%) offered primarily procedural assurances aligned with Lincoln & Guba (e.g., audio/video recording and verbatim transcription; protocol standardization; triangulation across interviews, observations, and documents; inductive/thematic coding by multiple trained researchers), with a small subset quantifying inter-coder agreement (Fleiss’ κ = 0.425–0.461; Kendall’s W = 0.489; both *p* < 0.001), indicative of moderate agreement by common benchmarks.

Validity reporting was even sparser: 30/42 (71.4%) psychometric instruments provided no study-context validity evidence. Among the remaining 12/42 (28.6%), evidence centred on structural validity (CFA/EFA/PCA), with fewer instances of convergent/discriminant/criterion and adaptation/procedural evidence. Most reported CFA solutions showed good-excellent fit (typically CFI ≈0.98–0.99, TLI ≈0.96–0.99, SRMR ≈0.02–0.03, RMSEA ≈0.04–0.07), though a minority were marginal (e.g., CFI ≈0.93; RMSEA ≈0.08). EFA/PCA findings commonly supported single-factor structures with high loadings, but KMO/Bartlett statistics and/or follow-up CFAs were frequently omitted. Convergent/criterion evidence included parallels with implicit and explicit indicators, age-appropriate known-group differences, and prediction of mathematics self-concept; discriminant evidence indicated separability from ability-stereotype measures.

Among the 23 studies that employed qualitative measurement tools, eight provided information regarding validity. The reported evidence was primarily grounded in content- and process-oriented indicators, including triangulation across field applications (observations, interviews, and materials), unannounced/randomized visits to capture typical lessons, video/audio recording and verbatim transcription, protocol standardization (shared opening prompt), thematic/iterative coding conducted by multiple researchers, prolonged engagement, and ethical safeguards (e.g., minimizing coercion, use of pseudonyms), as well as phenomenological reduction and context-specific descriptions. Notably, no quantitative validity evidence (e.g., convergent/discriminant/criterion relations, factor analysis, and measurement invariance) was identified in these studies; the reported indicators were confined to content and process assurances. Importantly, the κ/W values reported for expert agreement should be regarded as evidence of reliability, rather than validity.

### 3.6 Distribution of MGS-themed articles with regard to data analysis methods

**FIGURE 8:**
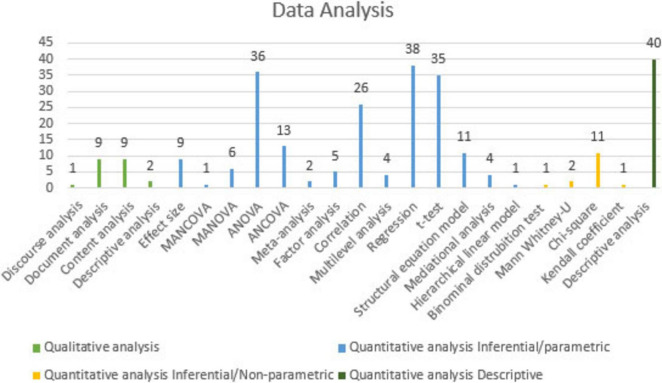
Distribution of data analysis methods in MGS-themed articles.

Figure 8 illustrates the distribution of data analysis methods used in MGS-themed articles.

Various data analysis methods have been used in articles themed on MGS. As seen in Figure 8, the most frequently used analysis method in quantitative studies is inferential/parametric analysis, which was employed in 191 studies. Among the inferential parametric analysis methods, regression analysis (38 studies), ANOVA (36 studies) and *t*-test (35 studies) stand out as the most commonly used methods. Additionally, correlation analysis (26 studies), and ANCOVA (13 studies) were also frequently preferred. The next most frequently used analysis method is statistical descriptive analysis, which was employed in 40 studies. Among the inferential non-parametric analysis methods, chi-square test (11 studies), Mann Whitney-U test (2 study), Kendall correlation coefficient (1 study), and binomial distribution test (1 study) were employed. For qualitative analysis methods, content analysis (9 studies), document analysis (9 studies), discourse analysis (1 study), and descriptive analysis (2 studies) were used.

### 3.7 Distribution of MGS-themed articles by the countries

**FIGURE 9:**
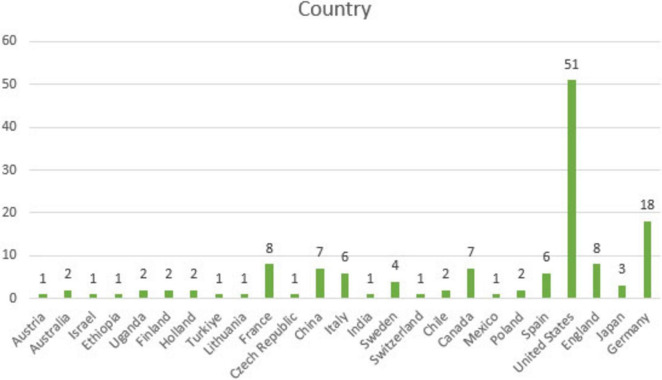
Distribution of countries represented in the reviewed MGS-themed articles.

Figure 9 displays the findings regarding the distribution of countries in the reviewed articles.

It is observed that a large proportion of articles themed on MGS have been published in Western countries. Countries such as the United States (51 studies) and Germany (18 studies) are prominent, while other Western countries like Spain (6 studies), the United Kingdom (8 studies), France (8 studies), and Italy (6 studies) also show a noticeable concentration. Other countries, on the other hand, host a relatively smaller number of studies. In the context of MGS research, contributions from Ethiopia (1 study), Uganda (1 study), India (1 study), Chile (2 studies), and Mexico (1 study) remain limited. This distribution suggests that the majority of studies are concentrated in the Western world, whereas regions such as the Global South contribute far less to the field. Accordingly, it may be argued that research on gender and mathematics in the Global South is still at an early, developmental stage.

### 3.8 Distribution of MGS-themed articles published over the years

**FIGURE 10:**
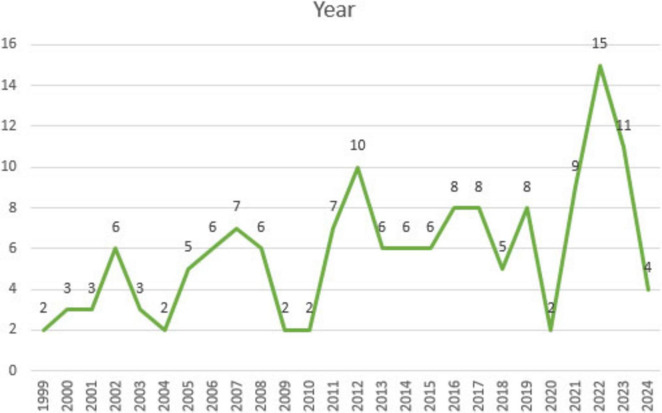
Annual distribution of publications on MGS.

Figure 10 illustrates the annual distribution of publications on MGS.

Figure 10 presents information on the distribution of the examined articles over the years. According to the data in the graph, there are noticeable fluctuations in the number of articles published on MGS. In 1999 the number of articles was limited to 2, while in 2002, it increased to 6. In 2012, however, there was a significant rise, reaching 10 articles. In 2013, 2014, and 2015, the number of articles remained around 6, while in 2016 and 2017, there was an increase, with eight articles published. In 2020, the number dropped further to 2. However, there was a resurgence in 2022, with seeing 15 articles. In 2023, the number reached 11, but in 2024, it dropped back to 4. These findings indicate that research in this area peaked particularly in 2012 and 2022, with fluctuations in other years.

### 3.9 Distribution of definitional axes of “stereotype” across the included articles

**FIGURE 11:**
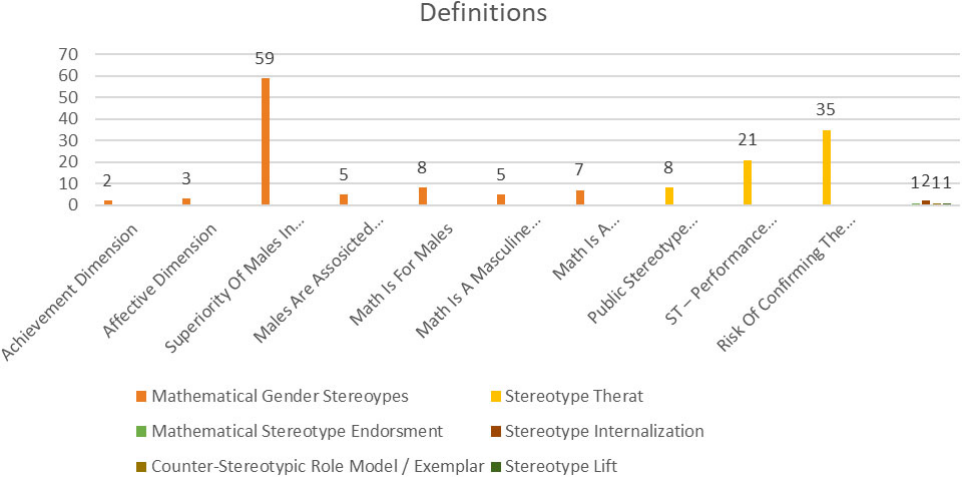
Distribution of definitional axes and subthemes across included studies.

Figure 11 illustrates the distribution of definitional axes and subthemes across the included studies.

Across the 158 definitional assignments, the literature conceptualizes stereotypes primarily along two axes: a belief/domain-ownership axis centered on male superiority, and a process-based axis centered on ST. The MGS (MGS) axis accounts for 89 (56.3%) of all instances; within MGS, Superiority of Males in Math is by far the most prevalent subtheme (*n* = 59; 66.3% of MGS). Additional MGS subthemes appear less frequently: Math Is for Males (*n* = 8; 9.0%), Math Is a Male/Gendered Domain (*n* = 7; 7.9%), Males Are Associated with Math (*n* = 5; 5.6%), Math Is a Masculine Domain (*n* = 5; 5.6%), Affective Dimension (*n* = 3; 3.4%), and Achievement Dimension (*n* = 2; 2.3%). The second major axis, ST (ST), comprises *n* = 64 (40.5%) of assignments. Here, Risk of Confirming the Stereotypes is most common (*n* = 35; 54.7% of ST), followed by ST—Performance Decrement (*n* = 21; 32.8%) and Public Stereotype Salience (*n* = 8; 12.5%). Other definitional axes collectively form a long tail (*n* = 5; 3.2%) including Stereotype Internalization (*n* = 2), Mathematical Stereotype Endorsement (*n* = 1), Counter-Stereotypic Role Model/Exemplar (*n* = 1), and Stereotype Lift (*n* = 1).

Consistent with the quantitative profile, the qualitative synthesis indicates a concentration of stereotypes along the belief/domain-ownership axis—particularly the emphasis on male superiority. Across many articles, the stereotype is framed as a direct belief in gender-based ability superiority. In our qualitative document analysis, multiple texts articulated this stance; one source states: “One of the most obvious forms of stereotyping relates to explicit beliefs alleging a male or female ability-superiority in domains such as mathematics and language arts” (Supplementary Appendix 1, Study 12, p. 2). In doing so, such texts position mathematical success and ability as more properly male, permeating both individual attitudes and contextual expectations. The most concrete instantiation of this theme reduces to the explicit claim that “boys are better at math” (Supplementary Appendix 1, Study 86, p. 597). This formulation recurred verbatim; for example, one text states: “gender stereotypes often manifest as the belief that boys are better at math than girls” (Supplementary Appendix 1, Study 142, p. 1). Together, these formulations show that superiority is articulated not only through implicit associations but also through explicit declarations, with implications for self-efficacy, sense of belonging, and expectancy structures.

In parallel, some studies do not state male superiority explicitly yet underscore the associative linkage between mathematics and masculinity; for example: “… stereotypical beliefs that associate math and gender (i.e., math-gender stereotypes, where math = male)” (Supplementary Appendix 1, Study 4, p. 638). Such associations function as an implicit filter for the questions “for whom” and “to whom it is suited,” thereby aligning domain belongingness with gender and reinforcing the psychosocial mechanisms noted above. Several texts go further, framing mathematics as male-owned rather than merely male-associated, marking a shift from belongingness to normative exclusion. A representative formulation states: “Mathematics-gender stereotype is the false idea that mathematics is for men, not for women” (Supplementary Appendix 1, Study 18, p. 123). These formulations legitimize institutional expectations that construct mathematics as a naturally male domain and, by implication, position girls as guests.

Additionally, several texts frame mathematical ability in essentialist, masculine terms—for example: “… mathematical ability’ as natural, individual and masculine…” (Supplementary Appendix 1, Study 88, p. 204), which portrays ability not as a developable skill but as an innate attribute aligned with masculinity. The corpus also frequently labels mathematics as a gendered domain; for example: “… stereotypically male domains such as mathematics” (Supplementary Appendix 1, Study 110, p. 233). Such usage constructs the field’s cultural image in a male-centered manner. Finally, some definitions extend beyond domain ownership/superiority to invoke affective (enjoyment/interest) and achievement (expectancies of success) dimensions. For instance: “It is a common stereotype that boys/men are more likely to enjoy and succeed in mathematics while girls/women are more likely to enjoy and succeed at language arts subjects that require more reading and writing skills” (Supplementary Appendix 1, Study 127, p. 173). These formulations reproduce the view that boys/men enjoy and excel in mathematics, whereas girls/women enjoy and excel in language-heavy subjects, thereby reinforcing gendered expectations in valuation, self-efficacy, and performance beliefs.

Mirroring the quantitative distribution, the qualitative synthesis shows a marked concentration along the process-based axis around the conceptualization of ST. First, across many texts, ST is framed as the risk of confirming a negative in-group stereotype. One source states this explicitly: “ST is the risk of confirming, as self-characteristic, a negative stereotype about one’s group” (Supplementary Appendix 1, Study 1, p. 62). Second—analogous to the superiority discourse—the most concrete outcome-level manifestation of ST is performance decrement; for example: “The threat of being negatively stereotyped in mathematics can impair the performance of women on difficult math tests, a phenomenon referred to as ST” (Supplementary Appendix 1, Study 151, p. 13). Third, some definitions—without invoking male superiority per se—emphasize public/social salience of the stereotype as the trigger of threat: “ST is the sense of threat that can arise when one knows that he or she can possibly be judged or treated negatively on the basis of a negative stereotype about one’s group” (Supplementary Appendix 1, Study 112, p. 437). Such framings suggest that expectations about who is doing the judging and by what criteria operate as an implicit filter, shaping perceptions of evaluative contexts and potentiating the threat experience.

Notwithstanding their small share (3.2%), these long-tail axes introduce distinct focal points that enrich the conceptual landscape and offer fine-grained process insights. In several reviewed studies, the emphasis shifts from situational activation to individuals’ agreement with or endorsement of the stereotype: “Specifically, mathematics-gender stereotype endorsement (MGS endorsement) regards the degree of agreement with or endorsement of this stereotype” (Supplementary Appendix 1, Study 18, p. 123). Other texts conceptualize the stereotype as a multi-stage process of internalization progressing from awareness to self-ascription: “Stereotype internalization is usually defined as the incorporation of negative societal views in the self-concept: People first become aware of societal stereotypes (e.g., their group reputation); then some of them tend to endorse these stereotypes (i.e., they believe the stereotype is true about their group); finally they come to internalize the stereotype believing that the stereotype is true about themselves” (Supplementary Appendix 1, Study 74, p. 858). This definition delineates a pathway awareness → endorsement → self-attribution, indicating durable effects on self-perception and suggesting interaction with—indeed, potential amplification of—stereotype-threat processes.

A further set of texts emphasizes the regulatory effect of information and figures that reverse the stereotypic association: “By definition, a counter-stereotype plays on stereotypes by reversing them. For Pedulla (2014), the central idea is that counter-stereotypical information provides positive associations between a perceiver and the negatively stereotyped individual or group” (Supplementary Appendix 1, Study 36, p. 4). Analytically, this framing posits positive associative bridges between the perceiver and the negatively stereotyped target, thereby weakening endorsement/internalization pathways and, contextually, attenuating the threat experience. Finally, some texts highlight a mechanism that operates asymmetrically within the same ecosystem: “stereotype lift,” which can be defined as “a performance boost that occurs when downward comparisons are made with a denigrated outgroup” (Supplementary Appendix 1, Study 1, p. 62). This indicates that downward social comparisons vis-à-vis a devalued out-group can yield performance gains—a process distinct from, yet mirroring, ST, with implications for how evaluative contexts distribute cognitive and motivational resources across groups.

Taken together, the corpus conceptualizes MGS predominantly through belief/domain-ownership formulations centered on male superiority and process-based formulations centered on ST. Less frequent, yet conceptually informative, are definitional strands concerning endorsement, internalization, counter-stereotypic exemplars, and stereotype lift. This layered map clarifies where the field’s definitional center of gravity lies and highlights under-articulated mechanisms that future research could leverage for theory development and intervention design.

### 3.10 Distribution of reported conclusions on MGS-themed articles

This section synthesizes the reported findings in the literature on MGS into six outcome categories: qualitative findings, descriptive findings, correlational findings, mediation findings, meta-analytic findings, and experimental findings.

**FIGURE 12:**
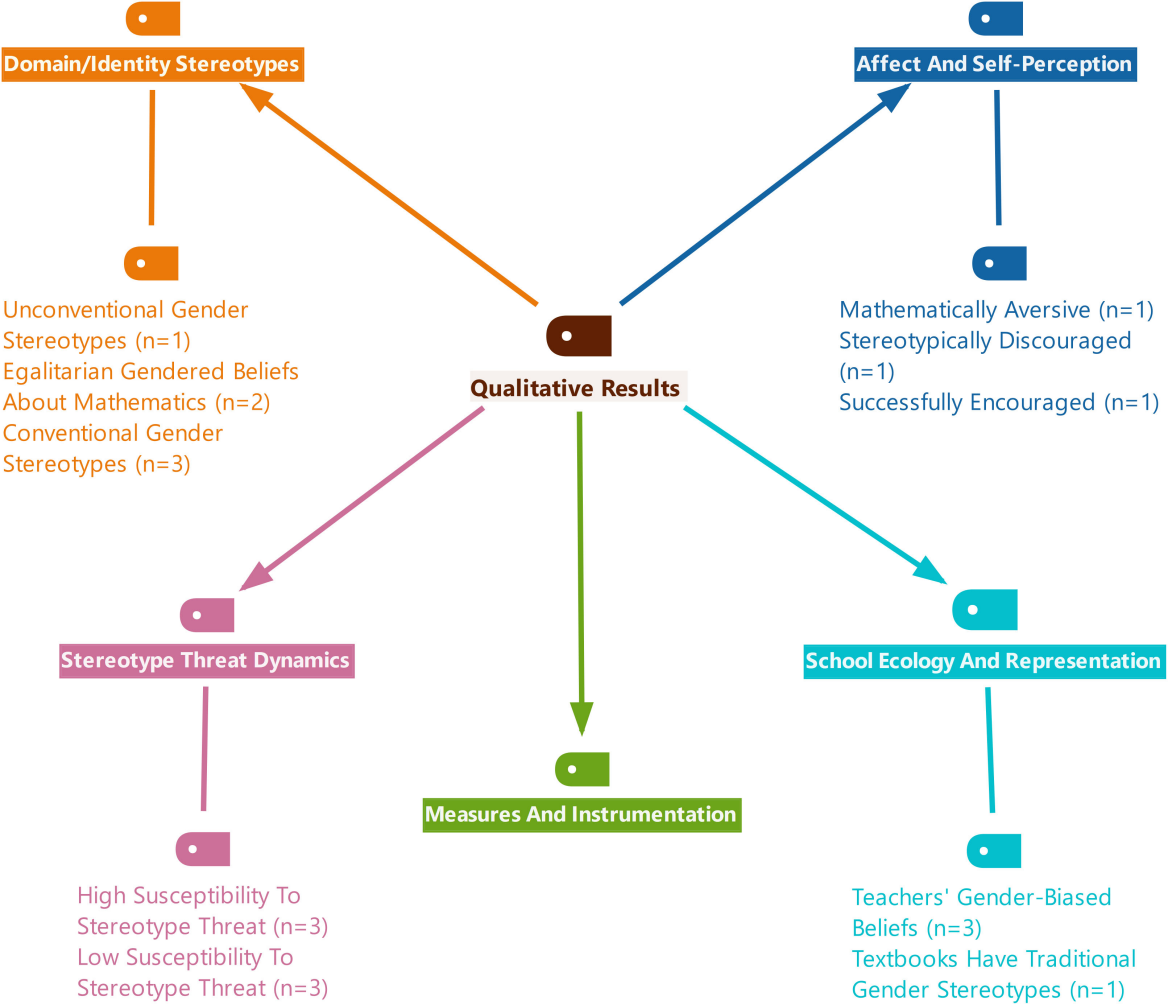
Thematic map of qualitative results in the reviewed articles.

Figure 12 displays the thematic map of qualitative results in the reviewed articles.

Qualitative findings converge around five themes: (i) School Ecology and Representation. One line of research documents how teacher discourse and material representation reproduce gendered expectations; for example, one study reports the following observation: “Everyone does the same exercises. The girls have a problem finding solutions by themselves…” (Supplementary Appendix 1, Study 63, p. 6). Another study examining imbalances in visual depictions arrives at this conclusion: “Altogether, of the 423 characters shown carrying out a professional activity, 146 are women, as compared to 277 men.” (Supplementary Appendix 1, Study 35, p. 1,491). (ii) Domain/Identity Stereotypes. A text documenting conventional framings states: “These discourses are oppositional and gendered; they inscribe mathematics as masculine…” (Supplementary Appendix 1, Study 88, p. 217). A study reporting more balanced cases notes: “It shows mostly balanced gender distribution in freetime and shopping categories…” (Supplementary Appendix 1, Study 38, p. 231). Another finding pointing to unconventional early-childhood patterns is conveyed as follows: “5-year-olds of both genders thought that girls liked math more than boys did” (Supplementary Appendix 1, Study 25, p. 1,273). (iii) ST Dynamics. Narratives differentiating by susceptibility record, for low susceptibility: “[In sixth grade] me and about four other people in our class were at a higher level than the rest of the class…” (Supplementary Appendix 1, Study 148, p. 614); and for high susceptibility, the same study offers: “Females who are good in math are smart… But males are probably gonna end up using math in their future…” (Supplementary Appendix 1, Study 148, p. 616). (iv) Measurement and Instrumentation. An example in which reliability is explicitly documented reports: “… Kendall’s *W* = 0.489, *p* < 0.001” (Supplementary Appendix 1, Study 17, p. 1). (v) Affect and Self-Perception. Among studies describing mathematically aversive profiles, one states: “These women do not like math…” (Supplementary Appendix 1, Study 105, p. 139); for successfully encouraged profiles, the same corpus reports: “These women appeared to have very positive attitudes toward math…” (Supplementary Appendix 1, Study 105, p. 136); stereotypically discouraged profiles are characterized as follows: “Two things are most noticeable about this group…” (Supplementary Appendix 1, Study 105, p. 139). Taken together, these findings indicate that mathematical gender stereotyping operates in a multi-layered manner across classroom ecology, domain-identity constructions, affective and self-belief processes, threat dynamics, and the quality of measurement.

**FIGURE 13:**
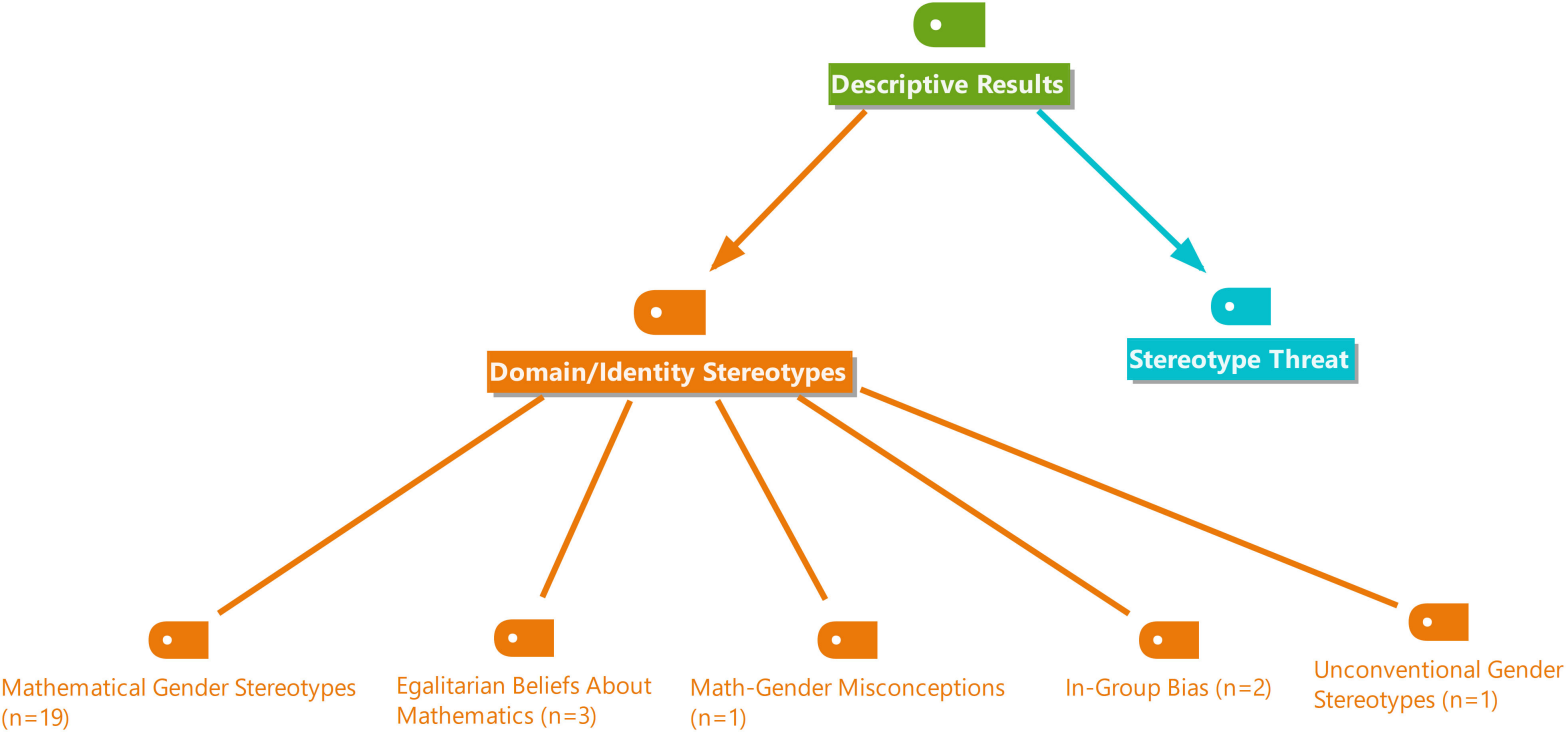
Higher-order themes in the descriptive strand of MGS literature.

In the descriptive strand of the literature, Figure 13 displays two higher-order themes: (i) Domain/Identity Stereotypes and (ii) ST. Codes cluster predominantly under the former. “MGS” (*n* = 19) is the dominant category; the masculinized framing of mathematics emerges early in development. “Egalitarian beliefs” (*n* = 3) point to more balanced representations. “Math-gender misconceptions” (*n* = 1) capture educator-specific misunderstandings. “In-group bias” (*n* = 2) and “unconventional” patterns (*n* = 1) are rare. Across descriptive studies, ST is reported only infrequently.

Within the correlational strand of the literature, the code co-occurrence network (Table 2) indicates that gender-stereotype endorsement (23 co-occurrences) and MGS beliefs (11) occupy central positions. The most frequent pairings are performance × stereotype endorsement (*n* = 8), performance × mathematical stereotype beliefs (*n* = 3), and academic intention × mathematical stereotype beliefs (*n* = 2). Math attitudes, self-ascribed ability, self-perception, participation, and math anxiety also co-occur primarily with stereotype endorsement. These analyses, however, do not establish causality; the co-occurrences reflect joint reporting within the same textual segments. The subsequent sections on moderation/mediation and experimental evidence elaborate the directionality of—and mechanisms underlying—these associations.

**TABLE 2:** Distribution of co-occurring codes in correlational studies on MGS.

Variables	Others	Counter-stereotypical role model	Mathematical gender stereotype beliefs	Men-math association	Gender differences in maths competencies	Gender stereotype endorsement
Others	3	0	1	0	1	0
Self-ascribed ability	0	0	0	0	0	1
Math attitudes	0	0	0	0	0	2
Rating their child mathematical competence	0	0	0	0	0	1
Beliefs about nature of mathematics	0	0	1	0	0	0
Maths anxiety	0	0	1	0	0	1
Mathematical performance	0	1	3	0	1	8
Academic Intention	0	0	2	0	1	1
Self-Doubt	0	0	1	0	1	0
Genetic determinism	0	0	1	0	0	1
Employment discrimination law	0	0	0	0	0	1
Math self-concept	0	0	1	0	0	2
Performance expectancies	0	0	0	0	0	1
Sense of fit in English	0	0	0	1	0	0
Participation	0	0	0	0	0	1
Attitude toward maths	0	0	0	0	0	1
Susceptible of stereotype threat	0	0	0	0	0	1
Self-perception	0	0	0	0	0	1
General gender related belief	0	0	0	0	1	0

Within the mediation strand of the literature, as summarized in Table 3, evidence from 11 SEM and 19 experimental mediation tests indicates that the dominant pathway links stereotypes to performance and intentions via self-beliefs and affect (e.g., self-concept, self-efficacy, anxiety). In two studies, parental math-gender stereotypes were associated with girls’ non-STEM orientations through intrusive support as a social-transmission mechanism. Consistent with this account, most experimental findings show that stereotype threat elevates anxiety, activates performance-avoidance goals, and elicits dejection, thereby undermining performance. By contrast, conditions incorporating self-affirmation/positive achievement identity, counter-stereotypic role models, or self-monitoring attenuate the threat effect and are associated with improved performance. A subset of studies reported null or mixed mediation effects.

**TABLE 3:** Pathways from stereotypes to outcomes: mediation results.

Study	Independent variable/predictor	Mediating variable	Dependent variable/outcome
SEM	Legitimatisation of gender difference status	Gender stereotype endorsement ↑	Mathematical competence ↓
SEM	Gender stereotype endorsement	Competence belief ↓/math value ↓	Career intention/mathematical performance ↓
SEM	Chronic stereotype threat	Intellectual helpless ↑/working memory ↓	Mathematical performance ↓
SEM	Parents gender stereotype endorsement	Girls’ motivation for language arts ↑	Academic intentions for STEM ↓
SEM	Mathematical gender stereotypes	Math interest	Mathematical performance ↓
SEM	Parents’ math-related gender stereotypes	Parents’ intrusive support ↑	Girls’ ability perception ↓
SEM	Mathematical gender stereotypes	Math self-concept ↑	Girls’ mathematical performance ↑
SEM	Mathematical gender stereotypes	Mathematics self-efficacy	Mathematics anxiety
SEM	Mathematical gender stereotypes	Mathematics self-efficacy	GPA for boys
SEM	Mathematical gender stereotypes	Mathematical self-concept/mathematics interest	Attitudes toward mathematics
SEM	Gender	Mathematics anxiety/mathematical gender stereotype endorsement	Mathematical performance
Experimental	Stereotype threat	Gender role orientation	Mathematical performance/mental rotation
Experimental	Stereotype threat	Intelligence	Mathematical performance
Experimental	Self-monitoring	Stereotype threat ↓	Females’ mathematical performance ↑
Experimental	Stereotype threat	Gender identity	Mathematical performance Ø
Experimental	Stereotype threat	Feminine identity ↓	Mathematics identity ↑
Experimental	Stereotype threat	Task absorption	Females’ performance-Avoidance Goals ↓
Experimental	Stereotype threat	Performance-avoidance goals/mathematics anxiety	Females’ mathematical performance ↓
Experimental	Stereotype threat	Positive achievement identity	Females’ mathematical performance ↑
Experimental	Stereotype threat	Self-evaluation math ability	Females’ mathematical performance ↓
Experimental	Stereotype threat	Mathematics anxiety	Females’ mathematical performance ↓
Experimental	Stereotype threat	Mathematical identity	Mathematical performance
Experimental	Stereotype threat	Positive role model	Females mathematical performance ↑
Experimental	Stereotype threat	Mathematical identity	Females mathematical performance ↑
Experimental	Stereotype threat	Being Latino	Females’ mathematical performance Ø
Experimental	Stereotype threat	Dejection emotions	Females’ mathematical performance ↓
Experimental	Stereotype threat	Mathematics identity	Mathematical performance Ø
Experimental	Stereotype threat	Mix-sex group	Females’ mathematical performance ↓
Experimental	Stereotype threat	Mathematics anxiety	Females’ mathematical performance ↓
Experimental	Stereotype threat	Self-control exertion	Mathematical performance Ø

Within the experimental strand of the literature, as summarized in Table 4, the modal pattern is that stereotype threat reduces women’s mathematics-related outcomes, whereas effects for men are typically null. The effect intensifies when the threat is made salient and in mixed-gender settings; conversely, conditions involving self-affirmation/positive achievement identity, counter-stereotypic role models, self-monitoring, and selected mindfulness practices attenuate—indeed, in some cases, reverse—the threat effect.

**TABLE 4:** Experimental manipulations and outcomes: direction of effects by gender and domain.

Manipulation	Domain effect	Direction of effect
Stereotype threat	Problem solving	Male Ø, Female ↓
Stereotype threat	Mental rotation performance	Female ↓
Stereotype lift	Mental rotation performance	Male ↓, Female ↑
Stereotype threat	Stereotype awareness	Male Ø, Female ↑
Stereotype threat	Mathematical performance	Female ↓
Stereotype threat/mindfulness intervention	Mental rotation performance	Male Ø, Female Ø
Stereotype threat	Mathematical performance	Female ↓
Stereotype threat	Mental rotation performance	Male ↑, Female ↓
Stereotype threat	Mathematical performance	Female ↓
Stereotype threat	Problem solving	Male Ø, Female ↑
Stereotype threat	Mathematical identity	Male ↑, Female ↓
Stereotype threat	Spatial orientation performance	Male ↑, Female Ø
Stereotype threat	Mathematical performance	Female ↑
Stereotype threat	Mathematical Performance	Male ↑
Stereotype threat	Spatial orientation performance	Male Ø, Female Ø
Stereotype threat	Mathematical Performance	Male ↑, Female ↓
Stereotype threat	Visuospatial ability	Male ↑, Female ↓
Stereotype threat	Mathematical performance/career intention	Female ↓
Stereotype threat	Problem solving	Female ↓
Stereotype threat	Mathematical performance	Female ↓
Stereotype threat	Mathematical performance	Male Ø, Female Ø
Stereotype threat	Physiological arousal	Female ↑
Stereotype threat	Mathematical performance	Female ↓
Stereotype threat	Mathematical performance	Female ↓
Stereotype threat	Mathematical performance	Female ↓
Stereotype lift	Mathematical performance	Female ↑
Stereotype threat	Mathematical performance	Female ↓
Stereotype threat	Performance avoidance	Female ↓
Stereotype threat	Mathematical performance	Female ↓
Stereotype threat	Mathematical performance	Female ↓
Stereotype threat	Mathematical performance	Female ↓
Stereotype threat	Neural networks	Female ↓
Stereotype threat	Mathematical performance	Female ↓
Stereotype threat	Mathematical performance	Female ↓
Stereotype threat	Mathematical performance	Female ↓
Identity threat model manipulation	Mathematical performance/self-esteem	Female ↑
Stereotype threat	Mathematical performance	Female ↓
Stereotype threat	Mathematical performance	Male ↑, Female ↓
Stereotype lift	Mathematical performance	Female ↑
Stereotype threat	Mental Rotation performance	Male ↑, Female ↓
Counter stereotypical manipulations	Mathematical performance	Female ↑
Counter stereotypical manipulations	Mathematical performance	Male Ø, Female Ø
Stereotype threat	Academic intention	Female Ø
Stereotype threat	Mathematical performance	Male Ø, Female Ø
Perspective-taking manipulations	Suppressing stereotypes about mathematics	Female Ø
Salience of system justification	Mathematical competence	Male ↑, Female ↓
Stereotype threat	Mathematical performance	Female Ø
Stereotype threat	Mathematical performance	Female Ø
Stereotype threat	Mathematical performance	Female ↓
Stereotype threat	Mathematical performance	Male Ø, Female Ø

Two comprehensive meta-analyses indicate that gender-ST is associated with small yet reliable performance decrements for girls/women. A child-adolescent meta-analysis (47 effects) estimated an average effect of d ≈-0.22, with no significant moderators and signals of publication bias. A broader synthesis (86 studies; 224 effects) found a small-to-moderate decrement only under threats targeting women (d ≈0.29), evident for mathematics but not for spatial tasks; heterogeneity was partly explained by task type, experimenter gender, and control condition. No consistent mean effects emerged for stereotype lift or for threats targeting men. Overall, the effects are context-sensitive and small in magnitude (| d| ≈0.20–0.30).

## 4 Discussion and Conclusion

This study presents the detailed distribution of MGS-themed articles by fields, topics, study groups, methods, data-collection instruments, data-analysis methods, countries, and years. In addition, the text discusses how the concept of “stereotype” is treated across definitional axes and how reported conclusions are distributed in MGS-themed studies; it also presents the distribution of instrument families and subtypes used in the reviewed research and offers a critical evaluation of the accompanying evidence on reliability and validity.

### 4.1 Field

The research findings indicate that the majority of studies are situated within the field of psychology. Additionally, a significant number of articles focused on MGS have been conducted in the fields of education and women’s studies. Given the concept of MGS, the emergence of this finding is quite natural.

Mathematical knowledge is often perceived as entirely rational (Tang et al., 2010); however, McDonald (1989) suggests that individuals have emotional responses to mathematics and that every thought has an emotional component. From this perspective, it can be argued that there is a relationship between emotions and information processing. It is well-established that individuals, particularly when faced with challenging learning experiences, experience emotions such as anxiety, which in turn shape their mathematical learning (Ashkenazi and Danan, 2017; Skagerlund et al., 2019). Additionally, attitudes and beliefs are significant factors that lead individuals to respond to mathematics in various ways. In this context, the impact of gender-based beliefs on mathematics is an important and noteworthy issue. Therefore, it is expected that studies related to MGS are frequently explored within the field of psychology.

Mandler (Adams and McLeod, 1989) posits that experiences lacking value or meaning do not elicit emotional responses. Accordingly, emotional reactions to mathematics may be understood as reflecting the cultural values to which individuals are exposed. For example, White students have frequently been observed to outperform their peers in mathematics (Brown-Jeffy, 2009), and in many countries boys score higher than girls (Ayalon and Livneh, 2013; Gutfleisch and Kogan, 2024). Indeed, the gender gap in mathematics achievement appears even more pronounced among Turkish-origin girls in the fourth grade, who occupy a “double-minority” position as the other within the other (Guiso et al., 2008). The relatively higher achievement of individuals from particular gendered and ethnic subcultures can be linked to the elevated value ascribed to science—particularly mathematics—since the Industrial Revolution, coupled with the pervasive belief that mathematics may not be suitable for everyone (McDonald, 1989). Within this frame, the causes and consequences of MGS naturally fall within the purview of researchers focused on gender equality.

Research indicates that male students outperform female students in cognitive domains that bear on academic achievement, such as problem solving and mathematical reasoning (Altunçekiç et al., 2005; Gallagher et al., 2000; Geary et al., 2000). In affective domains—mathematics anxiety, beliefs, self-confidence, self-efficacy, and attitudes toward mathematics—male students likewise tend to show more favorable outcomes than their female counterparts (Çakiroglu and Isiksal, 2009; Frenzel et al., 2007; Kargar et al., 2010; Keller, 2001; Köğce et al., 2009). Several studies directly examining academic performance in mathematics also report significant differences favoring male students (Tate, 1997; Van de Gaer et al., 2008). Nonetheless, perspectives positing a decisive biological basis for gender differences in mathematical ability (Auyeung et al., 2006) are challenged by evidence showing that studies of biological effects yield contradictory and insufficient results (Ceci et al., 2009). Caplan and Caplan (2005) argue that gender differences in mathematical ability have never been conclusively demonstrated and, when observed, are more plausibly attributable to factors linked to individual experiences. If biological differences do not necessarily exclude women from mathematics and mathematics-adjacent fields, then it is reasonable that researchers have shifted attention to classroom contexts to ask which experiences lead young women to disengage from mathematics (Keller, 2007). Accordingly, factors that may impede learning—such as MGS—have become a central focus for educators within a broad ecological framework spanning classroom practices, teacher attitudes, peer relations, and instructional materials. It follows that scholarship on mathematical gender roles has naturally moved from reductionist accounts emphasizing biological explanations of gender gaps in achievement toward research that centers educational practices.

As a result, it is important to explore MGS not only within the fields of psychology, education, and women’s studies but also across other related disciplines such as philosophy, sociology, communication, and science and technology. Moreover, such interdisciplinary research is expected to provide deeper insights from the perspective of gender equality.

### 4.2 Subject matters

The analysis reveals that “MGS” is the most frequently addressed topic in the reviewed literature, followed closely by “mathematical ST.” Other topics, such as “counter-stereotypical information about mathematical ability,” “gender equity in mathematics education,” and the “masculinity of mathematics,” are explored significantly less.

The prominence of MGS as the most frequently addressed topic in the reviewed literature is an expected outcome, given the scope of this systematic review. Since this study examines research on gender stereotypes in mathematics, the centrality of MGS aligns naturally with the thematic boundaries of the selected literature. Furthermore, this prevalence can be attributed to the foundational role the concept plays. It serves as a starting point for understanding and investigating related phenomena such as ST, counter-stereotype interventions, and gender inequalities in mathematics education.

Research examining how MGS shape individuals’ perceptions of mathematics (Martinot and Désert, 2007; Passolunghi et al., 2014; Tiedemann, 2002), career choice behaviors (Chaffee and Plante, 2022; Liu, 2018), and mathematical achievement (Cvencek et al., 2015; Smetackova, 2015; Song et al., 2016) has long been an important focus. Due to the widespread and profound impact of these stereotypes, it can be argued that they have become a central element in understanding gender inequalities in mathematics and fields requiring advanced mathematical skills. As a result, MGS have become a fundamental topic for both theoretical research and practical interventions.

In the reviewed studies it has been observed that the most frequently investigated topic after MGS is ST. ST can be defined as a psychological situation in which individuals are at risk of confirming a negative stereotype expectation based on their gender (Hyde et al., 2008). Comprehensive research on this threat not only aims to understand the existence of MGS but also explores the negative effects of these threats on the mathematical performance of stigmatized social groups (Hyde et al., 2008) and the psychological mechanisms involved in this process (Bertrams et al., 2022; Casad et al., 2017; Pérez-Garín et al., 2017). Specifically, this threat has been shown to have a significant negative impact on the academic performance of women, who are a group often questioned about their mathematical competence (Bedyńska et al., 2018; Doyle and Voyer, 2016). In this context, ST can be considered a significant reason for the challenges faced by girls and women in science, technology, engineering, and mathematics (STEM) fields. Therefore, research on ST clearly demonstrates that this issue is a critical topic and has been frequently addressed in the literature.

However, the intense emphasis on MGS and ST carries the risk of overshadowing other critical dimensions of the relationship between mathematics and gender. For instance, counter-stereotypical interventions that challenge traditional gender norms can provide valuable insights into mitigating the negative effects of these stereotypes. It is well-established that the presentation of female role models associated with mathematics and science reduces the harmful impacts of MGS (Drury et al., 2011). In a study conducted by Good et al. (2010), the effects of stereotypical (e.g., male scientists) and counter-stereotypical (e.g., female scientists) textbook visuals on high school students’ understanding of a science lesson were examined. The study concluded that female students demonstrated better comprehension when exposed to counter-stereotypical visuals.

Similarly, exploring the phenomenon of masculinity associated with mathematics offers a profound perspective on how male identities are constructed and how this influences students’ engagement in mathematics lessons. Studies reveal strong evidence that mathematics teachers pay more attention to male, students than female students and assign greater responsibilities to male students during the learning process. Furthermore, high-level cognitive questions are systematically directed at male students significantly more often than at female students (Mittelberg et al., 2011; Nurlu-Üstün and Aksoy, 2022). Male teachers are also noted to provide more support and attention to male students during problem-solving activities, and male students predominantly participate in mathematical discussions (Lafrance, 1991). Other research suggests that the increased interaction between teachers and male students places women at a clear disadvantage in mathematics lessons compared to their male counterparts (Black and Radovic, 2018).

A broader research agenda that includes these less-explored dimensions can help us grasp the complexities of gender dynamics in mathematics in a more nuanced manner. Future studies that go beyond focusing solely on MGS and ST could more effectively address the multiple and intersecting factors contributing to gender imbalances in mathematics and other related disciplines. Such an approach would foster a more comprehensive and holistic understanding of how gender operates in educational contexts and the broader professional world.

### 4.3 Research methods

Studies on MGS predominantly employ quantitative methods, with mixed and qualitative approaches being less commonly utilized. The prominence of quantitative research can be attributed to its inherent strengths. For instance, quantitative findings are often generalizable to entire populations or subpopulations due to their reliance on large, randomly selected samples (Carr, 1994). Additionally, the processes of data collection and analysis in quantitative research are typically time- and cost-efficient, often leveraging online surveys, forms, or statistical software.

Beyond these advantages, the dominance of quantitative research paradigms in this field can also be explained by the historical evolution and characteristics of the disciplines in which these studies are conducted. Most research on MGS has been carried out in psychology and educational sciences—fields that have historically favored quantitative approaches. This is consistent with findings in psychology (Gezici-Yalçın and Coskan, 2021) and educational sciences (Göktaş et al., 2012; Gül and Sözbilir, 2015; Nurlu-Üstün, 2023; Sağırlı-Özturan and Baş, 2020), where quantitative methodologies are predominant.

In psychology, this tendency is rooted in the discipline’s emergence as an independent science distinct from philosophy and medicine. From its inception, psychology adopted the deductive research methods of the natural sciences (Mayring, 2002). Early on, a dominant belief held that an objective reality existed independently of human perception or interpretation (Tebes, 2005). Consequently, experimental methods were advocated as fundamental to psychological research (Walsh et al., 2014). However, addressing complex societal issues such as MGS requires psychology to transcend these methodological limitations. There is a growing need for sophisticated approaches that integrate qualitative and mixed-methods research to provide a more nuanced understanding.

Similarly, educational research is also dominated by the quantitative paradigm. This prevalence is attributed to the scientific backgrounds of many education scholars, who often assume that research must produce statistical results, offer generalizable conclusions, and follow traditional methodologies (Ekiz, 2004). Yıldırım and Şimşek (2013) argue that some scholars trained in the positivist tradition reject qualitative methods that deviate from this framework as unscientific. Ekiz (2004) adds that these scholars have, either directly or indirectly, hindered the development and acceptance of qualitative methodologies in educational research. Nonetheless, studies exploring the impacts of MGS on educational settings, classroom practices, teacher behaviors, and student outcomes would benefit significantly from employing diverse qualitative research designs. Such approaches can offer a more comprehensive and holistic perspective, ultimately addressing critical gaps in the literature and advancing the field.

In conclusion, the predominance of quantitative methods in research on MGS stems from both the advantages of this methodology and the historical tendencies of disciplines such as psychology and educational sciences. However, relying solely on quantitative approaches may be insufficient to fully capture the complex social dynamics of this field. Therefore, future research should enhance methodological diversity to broaden the scope of the field and provide a more comprehensive perspective.

### 4.4 Sample

The research findings indicate that studies on MGS are most commonly conducted with undergraduate students, followed by middle and high school students. It has been observed that undergraduate students are the most common group sampled in studies on MGS. Similarly, Henrich et al. (2010) note that, particularly in the fields of psychology and cognitive science, samples are predominantly drawn from Western, Educated, Industrialized, Rich, and Democratic (WEIRD) populations, and more specifically from American undergraduate students. As Gül and Sözbilir (2015) have pointed out, conducting research with undergraduate students is often easier and more cost-effective.

The second most commonly studied groups are middle school students and high school students. Because the middle and high school years represent a critical period during which individuals’ career expectations begin to take shape and their vocational preferences are influenced (Correll, 2001). It is suggested that the initial steps of career planning are taken during this period (Göncü-Akbaş and Okutan, 2020). These years are regarded as a pivotal transition phase into either the workforce or higher education (Rowland, 2004). Consequently, the decisions made during this period play a significant role in shaping individuals’ vocational preferences in adulthood (Çakır, 2004). In this context, the increasing focus on middle and high school samples in studies examining the impact of MGS on career choices is noteworthy. The rise in research targeting these age groups can be considered a crucial step toward identifying stereotypes during this critical developmental period and developing intervention strategies. Such studies contribute significantly to the academic literature by facilitating the early detection and mitigation of negative stereotypes that could influence individuals’ career expectations.

However, it has been found that articles themed around MGS focus less frequently on elementary school students, preschool children, parents, teachers, and documents such as media elements and educational materials. One of the findings of this study is the limited number of studies conducted with samples from the preschool and elementary school years. The scarcity of studies may partly stem from the methodological difficulties of conducting survey-based research with children, including issues of comprehension and response validity. MGS weaken women’s and girls’ connection to mathematics, their sense of belonging (Good et al., 2010), and their willingness to engage in activities aimed at improving their mathematical abilities (Appel et al., 2011). This, in turn, negatively affects their mathematical achievement (Cvencek et al., 2015; Kiefer and Sekaquaptewa, 2007) and prevents them from pursuing careers that require advanced mathematical knowledge (Correll, 2001). Along with these negative effects of MGS, it is suggested that early ages are critical for the formation and reinforcement of such stereotypes (Cvencek et al., 2011; Del Río and Strasser, 2013; Herbert and Stipek, 2005). However, the limited number of studies conducted at the early childhood and elementary school levels indicates that gender stereotypes in these critical periods have not been sufficiently explored.

In addition, the limited focus on groups such as adults, teachers, and parents in studies on MGS may lead to a lack of comprehensive understanding of how these groups influence or are influenced by such stereotypes. It is well-established that both teachers and parents play a pivotal role in socializing children’s academic values and attitudes. A substantial body of research documents how parents’ and teachers’ expectations, gender stereotypes, and attributions affect children’s attitudes and performance in mathematics (Eccles et al., 1990; Ing, 2013; Tiedemann, 2000; Yee and Eccles, 1988). The limited research on groups such as adults, teachers, and parents regarding MGS results in a lack of understanding about how these groups influence or are influenced by such stereotypes. From this perspective, understanding how parents and teachers shape children’s perceptions of mathematics is crucial for developing interventions aimed at breaking these stereotypes.

In the literature on MGS, it has been observed that research based on documents is relatively scarce. However, studies that analyze documents such as textbooks and various media materials provide an important methodology for examining the historical and social context of MGS. For instance, textbooks, which are considered the primary source of knowledge (Çalışkan and Uymaz, 2022) and one of the most commonly used materials in classrooms (Kılıç and Seven, 2002), are distributed for free to students by the government in countries like Turkey. Therefore, these materials, which are accessible to every student, are shaped according to social and cultural norms and values (Wu et al., 2016). Zhang and Zhou (2008) emphasize that mathematics textbooks have a long-term and deep impact on students’ MGS, influencing their future mathematical learning processes. Studies have shown that while there is an attempt to maintain gender balance in mathematics textbooks, these materials still play a significant role in reproducing traditional gender stereotypes through elements such as occupational and family roles, as well as the gender of characters involved in mathematical activities (Guichot-Reina and De la Torre-Sierra, 2023; Moser and Hannover, 2014; Nurlu, 2021).

Similarly, media, ranging from movies to comic books and video games, has the power to convey stereotypical gender representations and mathematical content to new generations (Binark and Bek, 2009). Popular media tools aimed at children, including children’s books, television programs, films, websites, and video games, carry traditional gender stereotypes and contain gender-biased messages related to mathematics (Fellus et al., 2022; Hall and Suurtamm, 2020; Ladd, 2011; Wille et al., 2018). Similarly, posts on TikTok and X illustrate how the phrase “girl math” functions as a discursive practice. Within consumer and shopping contexts, the jargon frames women’s mathematical reasoning as “illogical” or “wrong.” Humorous examples circulate under this label, such as “Anything under five dollars feels like it’s pretty much free.” While presented as lighthearted or ironic, such usage reproduces the longstanding stereotype that girls and women lack mathematical competence. In this way, girl math normalizes gendered assumptions about cognitive ability and embeds them into everyday consumption practices and self-perceptions (Salma and Leiliyanti, 2024). Exposure to such media content has been shown to lead both male and female students to adopt these gender stereotypes (Hall and Suurtamm, 2020). In this context, studies on documents such as media materials and textbooks can provide valuable insights into how mathematical gender representations are shaped by societal norms and cultural values. However, the limited number of studies in this area suggests that the historical and cultural contexts have not been adequately addressed, highlighting the need for a broader perspective on MGS.

In conclusion, imbalances in research samples indicate the need for future studies to focus on a wider range of age groups and social roles. For instance, qualitative research on early childhood students and influential figures in their environment (such as parents and teachers), along with studies on documents such as educational materials and media elements, could provide a strong foundation for preventing and transforming MGS.

### 4.5 Data collection tools

These findings reveal that data collection tools in research on MGS are largely based on surveys and questionnaires. This can be seen as a reflection of the commonly preferred quantitative research methods in the literature and the generalizability advantages these methods provide. Quantitative data collection methods are frequently preferred because they are believed to yield high-quality data. These methods encourage more honest and sincere responses by ensuring anonymity and typically achieve higher response rates compared to methods like interviews. Additionally, surveys and questionnaires offer the ability to collect a large amount of data in a short period of time and at a low cost (Marshall, 2005).

However, the limited use of qualitative data collection methods such as interviews, observations, and document analysis in the studies examined creates a gap in the more in-depth and holistic exploration of the topic. This is because children and adults may be reluctant or unable to directly express their views on sensitive topics, such as gender stereotypes (Muzzatti and Agnoli, 2007). In this context, the interview method, which allows access to understanding another person’s perspective and gaining insight into their thoughts and stories (Patton, 2014), may enable a more comprehensive understanding of how MGS are shaped and propagated. Similarly, another important qualitative data collection method, observation, allows the researcher to experience the phenomenon first hand through direct observation rather than relying on assumptions. The observation method enables the researcher to engage directly with the environment, establish personal contacts, and gain a holistic understanding of the context in which individuals interact (Patton, 2014). In this regard, observation can be considered a valuable method for studying MGS. Moreover, document analysis, unlike data collected at the individual level, reflects our collective behaviors and reveals the dynamics at the societal level (Lune and Berg, 2017). In this context, document analysis can provide valuable insights into how social and cultural norms reinforce MGS. However, the widespread adoption of the quantitative paradigm in current research may limit the in-depth and qualitative understanding of societal phenomena such as MGS, thereby creating a significant narrowing in this area. Therefore, it should be considered that qualitative and mixed-method approaches could provide a more comprehensive perspective in understanding such complex social phenomena.

The widespread use of data collection tools categorized under the “other” category, such as ST manipulations, indicates a preference for behavioral and experimental approaches in research. These types of manipulations provide the opportunity to directly observe the effects of gender stereotypes, offering in-depth insights into how individuals respond to these stereotypes. However, it should be noted that these tools rely on a cause-and-effect relationship within a limited context, often conducted in laboratory settings, which raises questions about their applicability to persons, environments, treatments, and outcomes not included in the experiment (Shadish et al., 2002). In other words, findings from laboratory settings may not always be applicable to real-world contexts.

In conclusion, the imbalance in data collection tools points to the need for a more holistic approach to addressing MGS. Relying solely on quantitative data in research may be insufficient to understand the impact of stereotypes on individuals’ lives. Therefore, the use of qualitative data collection methods will contribute to a deeper understanding of these stereotypes and the development of more effective intervention strategies. Future studies should aim to balance both qualitative and quantitative research methods, enabling the generation of more comprehensive and accurate findings.

Our findings indicate that methodological transparency remains limited in both quantitative psychometric reporting and qualitative studies. On the quantitative side, only 23 of the 42 instruments identified across 152 studies (54.8%) reported reliability evidence, and most of these were confined to Cronbach’s α; 19 of 42 instruments (45.2%) provided no reliability information whatsoever. Yet the Standards for Educational and Psychological Testing (American Educational Research Association et al., 2014) frame the documentation of validity, reliability/measurement error (precision), and fairness for each intended use as a professional obligation. Likewise, APA JARS-Quant expects researchers to report study-specific reliability coefficients (e.g., internal consistency, test-retest, interrater agreement) alongside relevant validity evidence and implementation details aimed at improving measurement quality (Appelbaum et al., 2018). Methodological work further notes that the assumptions underlying α (tau-equivalence, independence of errors) are often violated, such that α may under- or over-estimate reliability; reporting McDonald’s ω in addition to α is therefore recommended (McNeish, 2017). On the validity side, 30 of the 42 instruments (71.4%) offered no context-specific evidence; the remaining 12/42 (28.6%) relied predominantly on structural validity (EFA/CFA/PCA). Although most CFA solutions reported good-excellent fit (CFI ≈0.98–0.99; TLI ≈0.96–0.99; SRMR ≈0.02–0.03; RMSEA ≈0.04–0.07), critical steps such as KMO/Bartlett diagnostics, parallel analysis, and independent confirmation were frequently omitted; convergent/discriminant/criterion and adaptation/procedural evidence was sparse. This pattern aligns with broader reviews showing that validity is either unreported or disproportionately reliant on structural indicators (Flake et al., 2017; Ntumi and Twum Antwi-Agyakwa, 2022). In such circumstances, the validity of research findings may be largely ungrounded and uninterpretable.

A similar picture emerges on the qualitative side. Only 23 of 152 studies (≈15.1%) employed qualitative techniques; of these, 18 (≈78.3%) did not report study-specific trustworthiness indicators. The remaining five (≈21.7%) provided process-based assurances aligned with Guba & Lincoln (recording-verbatim transcription, protocol standardization, triangulation, thematic coding by multiple researchers), while a small subset quantified inter-coder agreement at moderate levels (Fleiss’ κ = 0.425–0.461; Kendall’s *W* = 0.489; both *p* < 0.001). This pattern is consistent with work showing that qualitative reporting is typically at a moderate-low level and that “how trustworthiness was established” is often insufficiently specified (Walsh et al., 2020; Watts and Finkenstaedt-Quinn, 2021). Under the naturalistic paradigm, qualitative quality should be evaluated via credibility, transferability, dependability, and confirmability (Guba and Lincoln, 1985); however, these criteria should not be merely named. Following SRQR/COREQ/JARS-Qual, researchers should report in detail the definition and evidence of saturation, the nature of iteration, researcher positioning (reflexivity), and the scope of triangulation (O’Brien et al., 2014). Moreover, inter-rater reliability (IRR) is regarded as paramount in content analysis (Neuendorf, 2010); indeed, in science education journals, only 19 of 103 studies in 2019 reported IRR (Cheung and Tai, 2021). Process-based assurances are therefore necessary but not sufficient; where appropriate, they should be complemented by quantitative indices such as κ/α/AC1–AC2/W/ICC and reported transparently.

In sum, our results reveal that reporting on measurement quality remains limited in scope and depth across both quantitative and qualitative research. For policy and practice, we recommend: (i) reporting ω alongside α, as well as test-retest and ICC; (ii) supplementing structural validity with convergent/discriminant/criterion and adaptation/procedural evidence; and (iii) in qualitative studies, providing systematic and transparent accounts of reflexivity, saturation, iterative decisions, triangulation, and IRR. Such practices will strengthen the credibility, transferability, and dependability of findings.

### 4.6 Data analysis

This finding provides significant insight into the data analysis methods employed in research on MGS. Firstly, it is observed that the most frequently used methods in quantitative analyses are inferential analyses. This suggests that, whether employing experimental or survey designs, researchers aim to make inferences about variables and demonstrate how sample results can be generalized to a broader population (Creswell and Creswell, 2018). Additionally, it is noted that most of these inferential analyses are parametric, including ANOVA, *t*-tests, correlation, and regression analyses. The limited use of non-parametric analysis methods indicates a preference for parametric methods that typically validate their assumptions in research (Field, 2013). Chin and Lee (2008) state that parametric analyses offer more robust and reliable results compared to non-parametric ones. Therefore, the widespread use of parametric tests in articles focusing on MGS can be considered advantageous. Descriptive analyses play a significant role in studies focusing on MGS. The frequent use of descriptive analyses indicates that researchers often employ this method to define the group under study and summarize its characteristics using tables, graphs, and statistical measures such as central tendency and variability. This approach provides information about sample and population values (Çakıcı-Eser, 2022). Consequently, the application of descriptive statistics in articles addressing MGS has more clearly revealed the prevalence and acceptance rates of the phenomenon of mathematical gender stereotyping.

The observation that qualitative analysis methods—such as content analysis, document analysis, and discourse analysis—are less frequently employed compared to quantitative analyses is noteworthy. This trend may be attributed to the complexity and time-consuming nature of qualitative data analysis. Patton (2014) highlights these challenges, emphasizing the difficulty in reducing the volume of raw data, distinguishing the trivial from the significant, identifying key patterns, and constructing a framework that effectively conveys the essence of the data. However, in areas influenced by emotional and social factors, such as MGS, qualitative analyses can provide in-depth insights. They are instrumental in understanding how such stereotypes are shaped within social and cultural contexts. Therefore, these findings suggest that future research should aim for a more balanced application of both quantitative and qualitative data analysis methods.

### 4.7 Countries

The majority of academic research on MGS is concentrated in Western countries. Notably, the United States, Germany, France, and the United Kingdom are at the forefront of publications in this field, whereas academic studies on this topic are significantly less frequent in non-Western countries such as Israel, Ethiopia, Uganda, Turkey, and India. This disparity can be attributed not only to the overall dominance of Western countries in academic publishing but also to their higher levels of gender equality, democracy, and human rights.

The academic pre-eminence of Western nations is reinforced by historical processes, economic investments, and scientific publishing systems. From a historical standpoint, the Scientific Revolution in Central and Western Europe during the 16th and 17th centuries laid the foundation for the systematic production and dissemination of academic knowledge. For instance, in the 18th century, Encyclopédie, ouDictionnaireraisonné des sciences, des arts et des métiers, compiled by Diderot and d’Alembert, played a pivotal role in the structured development of knowledge by integrating scientific information within an interdisciplinary framework. Porter (1990) further asserts that the Scientific Revolution and the Enlightenment were instrumental in the emergence of modern social sciences such as sociology, economics, psychology, and anthropology. Thus, the quantitative dominance of academic output in Western countries has deep historical roots.

Moreover, the substantial financial resources allocated by Western nations for academic research and R and D (Research and Development) further solidify their leadership in scientific production. For example, annual R and D expenditures in the United States exceed $789 billion in 2021 (National Center for Science and Engineering Statistics, 2024). Such significant economic investments render the Western world an attractive hub for researchers, thereby facilitating brain drain from developing nations to more developed regions. Indeed, two-thirds of highly skilled immigrants have settled in North America (Lucas, 2008).

The Western-centric structure of scientific publishing systems further perpetuates this academic dominance. Academic reputation and influence are largely determined by journal rankings, impact factors, and H-indices. Established in 1963 with financial backing from the United States, the Science Citation Index (SCI) encompasses citations from the most prestigious scientific journals. However, the vast majority of these journals are based in the United States and the United Kingdom, while nearly all others originate from Europe. Over time, citation indices such as journal rankings have become key indicators of “reputable” academic knowledge, reinforcing a Euro-American-centered academic publishing landscape. Digitalization and financial investments have further amplified the impact of these citation indices, elevating Western academic networks to an even more dominant position. Consequently, regional academic journals and those publishing in languages other than English face increasing marginalization if they are not incorporated into these citation indices, thereby entrenching existing academic hierarchies. As a result, long-standing regional knowledge ecosystems are weakened, and the legitimacy of journals excluded from these indices is continuously scrutinized (Mills, 2024).

Another critical factor contributing to the concentration of academic studies on MGS in the West is the high level of development in gender equality, democracy, and human rights within these nations. According to the 2024 Global Gender Gap Report by the World Economic Forum, Europe has closed 75% of the gender gap, establishing itself as a global leader in this domain, while North America follows closely with a closure rate of 74.8%. In contrast, non-Western countries such as Israel, Ethiopia, India, and Turkey rank lower due to their comparatively weaker gender equality scores (World Economic Forum, 2024). Similarly, the 2024 Democracy Index classifies Europe and North America as “full democracies” (Economist Intelligence, 2025), while the Freedom in the World 2025 report by Freedom House designates countries in these regions as “free” (Freedom House, 2025). The presence of strong democratic institutions, extensive civil liberties, and robust human rights protections in these nations fosters a conducive environment for research on gender equality, thereby reinforcing the predominance of scholarly literature on this subject within Western academia.

While the academic dominance of Western countries is rooted in historical, economic, and structural factors, expanding research on MGS beyond these regions would enhance the diversity and inclusivity of knowledge production. To achieve this, fostering academic research in non-Western countries, integrating regional journals into international citation indices, and supporting scholars publishing in languages other than English are essential steps. Furthermore, advancements in gender equality, democracy, and human rights within these countries could create a more conducive environment for such studies. A more balanced global distribution of scientific knowledge would not only enrich academic discourse but also contribute to broader societal awareness and policy development.

### 4.8 Years

An examination of the annual distribution of research on MGS reveals fluctuations in the number of publications over time, with notable increases and decreases observed in specific periods. Since 1999, the number of articles has shown a fluctuating trend, with significant peaks in 2012 and 2022. In 2012, the number of publications reached its highest point with 10 articles, followed by a period of relative stability in 2017, when 8 articles were published. In 2022, the number of articles again reached 15, marking another peak. These fluctuations indicate that academic interest in the topic has concentrated in certain periods, influenced by various factors that may have shaped these trends.

The Fourth World Conference on Women, held in Beijing in 1995, significantly increased global awareness of gender equality and initiated a transformative process at the international level (United Nations, 2025). The Beijing Platform for Action, adopted at the end of the conference, facilitated the promotion of research on gender equality and pioneered the creation of international funding opportunities (UN Women, 2000). In this context, the emergence of academic studies on MGS from 1999 onwards can be seen as a consequence of the scientific and political environment shaped by the Beijing Platform for Action.

The increase in academic production in 2012 and 2022 can be attributed to global initiatives and policy changes aimed at advancing gender equality during these periods. Established in 2010, UN Women launched projects in 2011 to support the economic and academic empowerment of women, with various initiatives in the United States and the United Kingdom further complementing these efforts (UN Women, 2025). During the same period, the White House Council on Women and Girls in the U.S. allocated new funding to support women in STEM fields (White House Council on Women and Girls, 2025), while the National Science Foundation (NSF) expanded its ADVANCE Program to develop new policies aimed at empowering female academics (National Science Foundation, 2011). Similarly, in the UK, the Athena SWAN program was expanded to offer awards promoting gender equality in universities and research centers, providing support to female researchers (Athena SWAN, 2025).

The year 2022 stands out as a period during which academic funding agencies implemented stricter criteria for supporting gender equality. Under the European Union’s Horizon Europe Program, the Gender Equality Plan (GEP) became mandatory for research projects, and the inclusion of gender considerations in funding applications was established as an evaluation criterion (European Commission, 2022). Additionally, the “Women TechEU” program provided special funding for female leaders and entrepreneurs (European Innovation Council, 2025). UNESCO’s “STEM and Gender Advancement (SAGA)” initiative offered support to increase the participation of female academics in research, while UN Women and UNDP promoted gender equality awareness within academic institutions through the “Gender Equality Seal for Research Institutions” program (UNDP, 2025; UNESCO, 2025).

The fluctuations in academic interest in MGS at certain periods are directly linked to global policies, available funding, and academic trends. These dynamics help explain the periods of accelerated development in the field. However, the notable decline in the number of relevant publications in 2020, dropping to just two publications, necessitates an investigation into the underlying factors contributing to the fluctuations in scientific production.

Considering the impact of global crises on scientific output, the decline in these years becomes more understandable. For instance, the COVID-19 pandemic, which had a global impact in 2020, caused significant disruptions in academic research. The closure of universities, the transition to remote education, and the suspension of fieldwork greatly hindered scientific production. During this period, many scholars focused on the inequalities created by the pandemic in education (Czerniewicz et al., 2020; Frohn, 2021; Özer et al., 2020), and academic interest shifted toward educational technologies and remote teaching (Başaran et al., 2020). As a result, more specific areas, such as MGS, inevitably became a lower priority in academic agendas.

Moreover, the pandemic led to changes in the peer review and publication policies of many academic journals. As a result, a significant portion of the research published in 2020 focused on the COVID-19 pandemic (Raynaud et al., 2021; Riccaboni and Verginer, 2022). This shift contributed to the lower number of publications on MGS. Considering all these factors, the notable decline in publications in 2020 can be understood in the context of shifts in academic focus and the impact of global crises on scientific production.

The fluctuations in academic publications on MGS reflect broader global trends, shaped by political, social, and economic factors. While significant peaks in 2012 and 2022 highlight the importance of international initiatives and funding opportunities, the decline in 2020 suggest the vulnerability of research areas to shifts in academic focus and external crises, such as the COVID-19 pandemic. By recognizing and addressing the factors influencing these fluctuations, the academic community can better prioritize and continue to advance research on gender equality in mathematics.

### 4.9 Definitions

Our findings indicate that the literature organizes the theme of stereotypes along two robust axes: (i) belief- and belonging-based formulations centered on male superiority, and (ii) process-oriented formulations structured around ST. The former casts mathematics as a form of identity-linked “ownership,” shaping choice and persistence through belonging and expectancy structures; the latter attenuates momentary performance via cognitive and affective mechanisms activated within evaluative contexts. Together, these axes align with evidence on achievement disparities (Cvencek et al., 2015; Kiefer and Sekaquaptewa, 2007) and on problems of retention and persistence in the field (Correll, 2001).

Definitions that explicitly assert male superiority or implicitly construe mathematics as a “male domain” (Supplementary Appendix 1, Study 89, p. 237) emerge as the most persistent trope in the literature. This discourse structures not only individual expectancy beliefs but also classroom interaction patterns (Nurlu-Üstün and Aksoy, 2022), representational practices in instructional materials (Guichot-Reina and De la Torre-Sierra, 2023; Nurlu, 2021), and the visibility of role models (Ladd, 2011; Nurlu-Üstün and Uzuner-Yurt, 2023). Normative frames such as “Mathematics is for men, not for women” (Supplementary Appendix 1, Study 18, p. 123) masculinize the field as “naturally” male, thereby weakening girls’ sense of belonging and relegating them to a guest status (Good et al., 2012), and—over the longer term—suppressing course selection and career intentions (Correll, 2001).

Defining ST as the “risk of confirming, as self-characteristic, a negative stereotype about one’s group” (Supplementary Appendix 1, Study 1, p. 62) affirms the centrality of processes that are sensitive to evaluative context. As public visibility and the expectation of being judged increase, threat intensifies, producing performance decrements that are especially pronounced on cognitively demanding tasks (Steele and Aronson, 1995). This pattern helps explain why threat-reducing statements, low-threat task designs, and context-sensitive instructions can be effective.

Although the “long-tail” axes may appear quantitatively small, they enrich the stereotyping ecosystem. Endorsement and internalization delineate a pathway from mere awareness to self-ascription (McKown and Weinstein, 2003), whereas counter-stereotypic role models forge associative bridges that disrupt this chain (Dasgupta, 2011). Meanwhile, “stereotype lift” reminds us that derogating an out-group in comparative contexts can artificially inflate performance, underscoring the importance of fair assessment designs (Walton and Cohen, 2003).

The findings indicate that unidimensional measures may inadequately capture the plural nature of stereotypes. Future work should proceed with multidimensional scales that disentangle belonging/superiority, threat, endorsement, and internalization, and with designs that combine implicit and explicit indicators. At the same time, the conceptual map suggests that interventions must operate on two fronts: (i) representational and role-model strategies that weaken belonging/superiority discourse; and (ii) assessment designs and instructional guidelines that reduce the activation of threat.

### 4.10 Conclusions

Mathematics is widely framed as masculine; this framing is sustained by the school ecology (teacher discourse, material representation) and early-life experiences, and is transmitted to achievement and intentions via cognitive/affective processes. In the correlational network, stereotype endorsement and mathematical gender-stereotype beliefs occupy central positions; SEM and experimental evidence link them to performance through self-beliefs (self-concept/self-efficacy) and affective pathways (anxiety, dejection). This pattern accords with expectancy-value accounts (e.g., self-concept/self-efficacy and domain value (Eccles and Wigfield, 2020) and with stereotype-threat theory (Steele, 1997): gendered contexts erode self-resources, activate avoidance goals, and depress performance.

Meta-analytic evidence indicates small but reliable effects (approximately | d| ≈0.20–0.30) that are sensitive to context. Effects tend to intensify when threat is salient and in mixed-gender settings. However, they can be attenuated—and in some cases even reversed—through self-affirmation/positive achievement identity, counter-stereotypic role models, self-monitoring, and selected mindfulness practices. Experimental mediation findings largely align with a “threat → anxiety/avoidance → performance ↓” pathway (Schmader et al., 2004). Nevertheless, some studies report null or complex mediation, suggesting that task type, measurement timing, and differences in operationalization are decisive factors (Pennington et al., 2016).

Linking parental stereotypes—enacted through behaviors such as intrusive support—to girls’ non-STEM trajectories indicates that stereotypes function as social practices transmitted within the family-school ecology, rather than merely as individual attitudes (Carlana, 2019; Crowley et al., 2001). Likewise, teacher discourse and the uneven representation in instructional materials generate symbolic cues that, as qualitative excerpts illustrate, entrench classroom norms (Blumberg, 2008; Tiedemann, 2002).

Taken together, these findings underscore both conceptual and methodological challenges, while simultaneously pointing toward actionable implications for practice and future research. Methodologically, divergent operationalization of “mathematics-gender stereotype” (identity labels, competence clichés, implicit associations) hinder cross-study comparability. Although some reliability is reported, stronger evidence is needed for measurement invariance across age and cultural groups and for adherence to reporting standards. Empirically, the findings support: (i) teacher professional development (language/comparative feedback), (ii) curriculum-materials review (balanced representation), (iii) pre-exam self-affirmation/identity-supportive micro-interventions, (iv) visibility of counter-stereotypic role models, and (v) adjustments to class composition and task framing—each requiring context-sensitive design and external-validity testing. Priorities for future work include adequately powered, multi-source experiments; time-segmented mediation and multilevel SEM; rigorous measurement invariance by age/sex/culture; standardized operationalization of threat salience, control conditions, and task types; and open-science practices. Longitudinal studies of early-childhood masculinized framings could identify ecological windows for intervention.

The evidence demonstrates that MGS operate in multilayered ways, with effects that are small yet consistent and context-sensitive, and that these effects can be mitigated through appropriate psychosocial interventions and ecological adjustments. Advancing definitional and measurement standardization, alongside strengthening causal research designs, appears critical for the next advancement of the field.

## 5 Conclusion

The findings of this review indicate that research on MGS is concentrated primarily within psychology, followed by education/educational sciences and women’s studies, with only limited contributions from other fields such as communication or science and technology. Thematically, the most frequently examined subject is MGS themselves, with related strands including ST, counter-stereotypical information, the masculinity of mathematics, and gender equity in mathematics education. Methodologically, studies are predominantly quantitative, with experimental designs most common, followed by surveys and scale development, while qualitative and mixed-method approaches remain scarce. Data are largely collected from undergraduate students, with fewer studies focusing on other populations such as school-aged children, teachers, parents, or early childhood groups. Surveys and questionnaires dominate as data collection instruments, supplemented by stereotype-threat manipulations, whereas qualitative tools such as interviews and observations are rarely employed. Analytically, the literature relies heavily on inferential parametric statistics—particularly regression, ANOVA, and *t*-tests—while descriptive and non-parametric analyses are used less frequently, and qualitative methods are underrepresented. Geographically, research is concentrated in Western countries, especially the United States and Germany, with only sparse contributions from the Global South, where such scholarship appears still emergent. Publication trends fluctuate over time, with peaks in 2012 and 2022. Conceptually, stereotypes are defined along two main axes: belief/domain-ownership formulations centered on male superiority and process-based formulations centered on ST, with less frequent but conceptually meaningful extensions such as endorsement, internalization, counter-stereotypic role models, and stereotype lift. Finally, across six outcome categories—qualitative, descriptive, correlational, mediation, meta-analytic, and experimental—findings converge to show that stereotypes operate primarily through self-beliefs and affective processes, influencing performance and intentions, with effects that are small yet reliable, context-sensitive, and attenuated by interventions such as self-affirmation, counter-stereotypic role models, and self-monitoring.

### 5.1 Limitations

This systematic review has several limitations that should be acknowledged. First, the literature search was restricted to the Web of Science database. Although Web of Science indexes high-quality, peer-reviewed publications, the exclusion of other major databases (e.g., Scopus, ERIC, PsycINFO) may have led to the omission of relevant studies—particularly education-sector proceedings, regional journals, and psychology-specific outlets. This coverage decision could bias the corpus toward internationally indexed journals and may have affected the observed distribution of samples (e.g., early childhood/elementary), methods, and contexts. Future updates should implement multi-database searches (WoS, Scopus, ERIC, PsycINFO), and backward/forward citation chasing to strengthen comprehensiveness and reproducibility.

Second, the review included only studies published in English, potentially excluding valuable research conducted in other languages—particularly in non-Western contexts. This language restriction may have limited the cultural and geographical diversity of the findings.

Third, gray literature such as dissertations, conference proceedings, and institutional reports was not included in the review. As a result, emerging or unpublished research related to MGS may have been overlooked.

No protocol pre-registration (e.g., PROSPERO/OSF) was undertaken for this systematic review. The absence of pre-registration constitutes a limitation, as it may increase the flexibility of decision-making during the study and thereby heighten the risk of selection or reporting bias. To mitigate this risk, however, the inclusion/exclusion criteria and the analysis plan were specified in writing prior to the search, and adherence to the PRISMA 2020 guidelines was maintained (Supplementary Appendix 2).

Finally, although the coding procedure followed a systematic approach and inter-coder reliability was established, some degree of subjectivity in interpreting and categorizing studies is unavoidable. In addition, the review was conducted by a single researcher; the absence of independent dual screening and cross-validation constitutes an additional limitation that may increase the risk of selection and reporting bias. Taken together, these factors suggest that the synthesis may inadvertently reflect some bias. A list of PRISMA items not implemented and the justification for their omission is available in Supplementary Appendix 3.

## Data Availability

The original contributions presented in the study are included in the article/Supplementary material, further inquiries can be directed to the corresponding author.
