# Supplementary material for: Unpacking mathematical gender stereotypes: trends and directions from 25 years of research
Source: Front Psychol. 2025 Nov 18;16:1660583. doi: 10.3389/fpsyg.2025.1660583 (PMC12669155; doi:10.3389/fpsyg.2025.1660583)
Supplement: Supplementary file 1 [file Supplementary_file_1.docx]

| S1 Appendix. List of the Analysed Articles Included in the Systematic Review | | | |
| --- | --- | --- | --- |
|  |  |  |  |
| **No** | **Title** | **Autho(s)** | **Year** |
| 1 | A Threat In The Classroom  Gender Stereotype Activation And Mental-Rotation ​Performance In Elementary-School Children | Sarah Neuburger, Petra Jansen, Martin Heil, Claudia Quaiser-Pohl | 2012 |
| 2 | Stereotype Threat Effects On Italian Girls’ Mathematics Performance: ​A Failure To Replicate | Franca Agnoli, Francesca Melchiorre, Claudio Zandonella Callegher, Gianmarco Altoè | 2021 |
| 3 | Cognitive, Educational And Psychological ​Determinants Of Prospective Preschool Teachers’ ​Beliefs | Sigrid Blömeke, Simone Dunekacke, Lars Jenßen | 2017 |
| 4 | Early Sources Of Children’s Math Achievement In Chile: The Role Of ​​Parental Beliefs And Feelings About Math | M. Francisca del Río, María Inés Susperreguy, Katherine Strasser, Dario Cvencek, Carolina Iturra, Ismael Gallardo, Andrew N. Meltzof | 2021 |
| 5 | Masculinity In The Public Image ​​Of Physics And Mathematics: ​​A New Model Comparing Japan ​​And England | Yuko Ikkatai, Atsushi Inoue, Azusa Minamizaki, Kei Kano, Euan McKay, Hiromi M. Yokoyama | 2021 |
| 6 | Urban Elementary ​​Single-Sex Math ​ Classrooms: Mitigating ​​Stereotype Threat For ​​African American Girls | Anica G. Bowe, Christopher D. Desjardins, Lesa M. Covington Clarkson, Frances Lawrenz | 2017 |
| 7 | Students' Gendered Perceptions Of Mathematics In Middle Grades ​​Single‐Sex And Coeducational Classrooms | Dennis Kombe, William Bridges, S. Megan Che | 2019 |
| 8 | Mathematics Self-Concept And Response Pattern ​In Higher Education Examinations: Differences ​Between Genders | María Isabel Núñez-Peña, Marta Ramon-Casas, Toni Cunillera & Carlos ​Campos-Rodríguez | 2024 |
| 9 | Self-Control Capacity Moderates The ​​Effect Of Stereotype Threat On ​​Female University Students’ Worry ​​During A Math Performance Situation | Alex Bertrams, Christoph Lindner, Francesca Muntoni, Jan Retelsdorf | 2022 |
| 10 | Measuring Stereotype Threat At Math  And Language Arts In Secondary  School: Validation Of A Questionnaire | Sylwia Bedynska, Piotr Rycielski, Magdalena Jabłonska | 2021 |
| 11 | A Study On The Influence Of The Affective Domain On The Attitudes Of Middle School Students Toward Mathematics From A Gender Perspective | M Gutierrez-Aguilar, S Tejeda | 2024 |
| 12 | How Parents’ Stereotypical Beliefs ​Relate To Students’ Motivation And ​Career Aspirations In Mathematics ​And Language Arts | Kathryn Everhart Chaffee, Isabelle Plante | 2022 |
| 13 | Divergent Effects Of System ​​Justification Salience On The ​​Academic Self-Assessments Of ​​Men And Women | Virginie Bonnot, John T. Jost | 2014 |
| 14 | Race, Gender, And Teacher Equity Beliefs: Construct Validation ​​Of The Attributions Of Mathematical Excellence Scale | Erik Jacobson, Dionne Cross Francis, Craig Willey, Kerrie Wilkins-Yel | 2022 |
| 15 | The Effect of the Interplay of Gender and Ethnicity on Teachers Judgements: Does the School Subject Matter? | Meike Bonefeld, Hannah Kleen, and Sabine Glock | 2022 |
| 16 | Investigating Classroom Implementation Of Research-Based ​Interventions For Reducing Stereotype Threat In Calculus | Elizabeth G. Arnold, Elizabeth A. Burroughs, Jessica M. Deshler | 2020 |
| 17 | Design And Validation Of A Classroom Observation Instrument ​To Evaluate The Quality Of Mathematical Activity From A ​Gender Perspective | Lorena Espinoza Salfate, Gonzalo Guerrero, Joaquim Barbé Farré, Felipe Márquez Salinas | 2023 |
| 18 | Mathematics–Gender Stereotype Endorsement Influences ​Mathematics Anxiety, Self-Concept, And Performance ​Differently In Men And Women | Serena Rossi, Iro Xenidou-Dervou, Emine Simsek, Christina Artemenko, Gabriella Daroczy,Hans-Christoph Nuerk, Krzysztof Cipora | 2022 |
| 19 | Stereotype Threat May Not Impact Women’s Inhibitory Control Or ​Mathematical Performance: Providing Support For The Null ​Hypothesis | Charlotte R. Pennington, Damien Litchfield, Neil McLatchie, Derek Heim | 2019 |
| 20 | Creating A Critical Mass Eliminates The Effects Of ​Stereotype Threat On Women’s Mathematical ​Performance | Charlotte R. Pennington, Derek Heim | 2016 |
| 21 | Toward Gender Equality In Education—Teachers’ Beliefs About ​Gender And Math | Jana Lindner, Elena Makarova, Deborah Bernhard, Dorothee Brovelli | 2022 |
| 22 | ‘Boys Press All The Buttons And Hope It Will Help’: ​Upper Secondary School Teachers’ Gendered ​Conceptions About Students’ Mathematical Reasoning | Lovisa Sumpter | 2016 |
| 23 | Being Inclusive Or Reinforcing Of Social Stereotypes The Case Of Kerala State Board Mathematics Textbooks | Jayasree Subramanian, Anagha S | 2023 |
| 24 | Stereotype Threat, Gender And Mathematics ​Attainment: A Conceptual Replication Of ​Stricker & Ward | Matthew Inglis, Steven O’HaganI | 2022 |
| 25 | Preschoolers’ Mathematical Play And Colour ​Preferences: A New Window Into The Development ​Of Gendered Beliefs About Math | Jesús Paz-Albo Prieto, Dario Cvencek, Cristina V. Herranz Llácer, Aránzazu ​Hervás Escobar, Andrew N. Meltzoff | 2017 |
| 26 | What Does Gender Has To Do With Math? Complex Questions ​​Require Complex Answers | Luisa Girelli | 2023 |
| 27 | K-8 Teachers’ Overall And Gender-Specific Beliefs ​About Mathematical Aptitude | Yasemin Copur-Gencturk, Ian Thacker, David Quinn | 2021 |
| 28 | The Role Of Perspective-Taking In Suppressing ​​Stereotypes About Mathematics | Mana Yamamoto, Takashi Oka | 2023 |
| 29 | Stereotype Threat And Gender: Math ​​Performance In Chinese College Students | Ming Tsui, Xiao-ying Xu, Edmond Venator,Yan Wang, | 2016 |
| 30 | Stereotype Manipulation Effects On Math And Spatial Test Performance: ​A Meta-Analysis | Randi A. Doyle, Daniel Voyer | 2016 |
| 31 | Gender Stereotypes: Implicit Threat To Performance ​​Or Boost For Motivational Aspects In Primary School? | Johanna Maria Hermann, Regina Vollmeyer | 2022 |
| 32 | Gender Stereotypes, Performance And Identification With Math | Irena Smetackova | 2015 |
| 33 | Parent-Child Math Anxiety And Math-Gender Stereotypes Predict Adolescents’ Math Education Outcomes | Bettina J. Casad, Patricia Hale, Faye L. Wachs | 2015 |
| 34 | Stereotype Threat Among Girls: ​Differences By Gender Identity ​And Math Education Context | Bettina J. Casad, Patricia Hale, Faye L. Wachs | 2017 |
| 35 | The Representation Of Gender Stereotypes In Spanish ​​Mathematics Textbooks For Elementary Education | Virginia Guichot-Reina, Ana María De la Torre-Sierra | 2023 |
| 36 | Counter-Stereotypes And Images: An Exploratory ​Research And Some Questions | Christine Morin-Messabel, Se´verine Ferrie`re, Frederic Martinez, Julie Devif, Laurence Reeb | 2017 |
| 37 | Can Gender Priming Eliminate The Effects Of Stereotype Threat? The Case Of ​Simple Dynamic Systems | Vivien Lungwitz, Peter Sedlmeier, Marcus Schwarz | 2018 |
| 38 | Gender Representation In The National ​​Assessments Of Mathematical ​​Achievements | Monika Grigaliūnienė, Roma Kačinskaitė | 2021 |
| 39 | Chronic Stereotype Threat Is ​Associated With Mathematical ​Achievement On Representative ​Sample Of Secondary Schoolgirls: ​The Role Of Gender Identification, ​Working Memory, And Intellectual ​Helplessness | Sylwia Bedynska, Izabela Krejtz, Grzegorz Sedek | 2018 |
| 40 | Exploring The Nature Of Teachers’ ​Math-Gender Stereotypes: The ​Math-Gender Misconception ​Questionnaire | Anna-Sophia Dersch, Anke Heyder, Alexander Eitel | 2022 |
| 41 | Implicit Math-Gender Stereotype Present In Adults But Not In 8th ​Grade | Kyle Morrissey, Darcy Hallett, Aishah Bakhtiar, Cheryll Fitzpatrick | 2019 |
| 42 | Can Math-Gender Stereotypes Be Reduced? A Theory-Based ​Intervention Program With Adolescent Girls | Fengqing Zhao, Yiyin Zhang, Valeria Alterman, Baoshan Zhang, Guoliang Yu | 2018 |
| 43 | Gender In Mathematics: How Gender Role ​​Perception Infuences Mathematical Capability ​​In Junior High School | Guihua Xie, Xinyu Liu | 2023 |
| 44 | Determination Of Primary School Teachers' ​​Mathematical Gender Stereotypes And ​​Examination Of Their Reflection On Students | Ozge Nurlu Ustun, Naciye Aksoy | 2022 |
| 45 | Gender‑Math Stereotypes And Mathematical Performance: ​​The Role Of Attitude Toward Mathematics And Math ​​Self‑Concept | Fang Xie, Yan Yang, Cong Xiao | 2023 |
| 46 | Gender Differences In Young Adults' Mathematical Performance: Examining ​​The Contribution Of Working Memory, Math Anxiety And ​​Gender-Related Stereotypes | Helene Vos, Mila Marinova, Sara C. De L´eon, Delphine Sasanguie, Bert Reynvoet | 2023 |
| 47 | Variations Of Gender–Math Stereotype Content Affect ​Women’s Vulnerability To Stereotype Threat | Dustin B. Thoman, Paul H. White, Niwako Yamawaki, Hirofumi Koishi | 2008 |
| 48 | Are Parents’ Academic Gender Stereotypes and ​Changes in Them Related to Their Perceptions of ​Their Child’s Mathematical Competence? | Hannu Räty, Riitta Kärkkäinen | 2011 |
| 49 | Mind The Gap: Framing Of Women’s Success And Representation ​In STEM Affects Women’s Math Performance Under Threat | Emily S. Shaffer, David M. Marx, Radmila Prislin | 2013 |
| 50 | Gender Differences In Persistence And Attributions ​In Stereotype Relevant Contexts | Amy Kiefer, Margaret Shih | 2006 |
| 51 | Effects Of Salient Multiple Identities On Women’s ​Performance Under Mathematics Stereotype Threat | Dana M. Gresky, Laura L. Ten Eyck, Charles G. Lord, Rusty B. McIntyre | 2005 |
| 52 | Understanding The Paradox In Math-Related Fields: Why Do ​Some Gender Gaps Remain While Others Do Not? | Sapna Cheryan | 2012 |
| 53 | Culture, Context And Stereotype Threat: A ​Comparative Analysis Of Young Ugandan Women ​In Coed And Single-Sex Schools | Katherine Picho, Jason M. Stephens | 2012 |
| 54 | In¯ Uence Of Item Content And Stereotype Situation ​On Gender Differences In Mathematical ​Problem Solving | Margaret Walsh, Crystal Hickey, Jim Duffy | 1999 |
| 55 | Gender Stereotype Endorsement And Achievement-Related Outcomes: ​The Role Of Competence Beliefs And Task Values | Isabelle Plante, Roxane de la Sablonnière, Joshua M. Aronson, Manon Théorêt | 2013 |
| 56 | The Effects Of Stereotype Threat And ​Double-Minority Status On The Test ​Performance Of Latino Women | Patricia M. Gonzales ​Hart Blanton ​Kevin J. Williams | 2002 |
| 57 | Reducing Stereotype Threat In Order To Facilitate Learning | Kathryn L. Boucher, Robert J. Rydell, Katie J. Van Loo, Michael T. Rydell | 2012 |
| 58 | Girls’ Performance In Mathematics ​In Upper Primary Schools Of ​Addis Ababa | Tilaye Kassahun, Bedru Kedir | 2006 |
| 59 | Parents’ Explanations Of Their Child’s Performance ​In Mathematics And Reading: A Replication ​And Extension Of Yee And Eccles | Hannu Raty, Johanna Vansk, Kati Kasanen, Riitta Karkkainen | 2002 |
| 60 | Stereotype Threat In The Classroom: ​Dejection Mediates The Disrupting Threat ​Effect On Women’s Math Performance | Johannes Keller, ​Dirk Dauenheimer | 2003 |
| 61 | Stereotype Threat Among School Girls In Quasi-Ordinary Classroom ​Circumstances. | Pascal Huguet, Isabelle Re´gner | 2007 |
| 62 | Stereotype Internalization, Math Perceptions, And Occupational Choices Of ​Women With Counter-Stereotypical University Majors | Virginie Bonnot, Jean-Claude Croizet | 2007 |
| 63 | Mathematics And Gender Stereotypes In  One Jewish And One Druze Grade 5 Classroom In Israel | David Mittelberg, Osnat Rozner, Helen Forgasz | 2011 |
| 64 | An Examination Of Implicitly Activated, Explicitly ​Activated, And Nullified Stereotypes On Mathematical ​Performance: It’s Not Just A Woman’s Issue | Jessi L. Smith, Paul H. White | 2002 |
| 65 | The Negative Consequences ​Of Threat | Anne C. Krendl, Jennifer A. Richeson, William M. Kelley, Todd F. Heatherton | 2008 |
| 66 | Reducing The Impact Of Stereotype Threat On Women's Math Performance: Are Two  Strategies Better Than One? | Paul R. Jones | 2011 |
| 67 | Discounting The Difficult: How High Math-Identified ​Women Respond To Stereotype Threat | Alexandra C. Lesko, Jennifer Henderlong Corpus | 2006 |
| 68 | French Children’s Awareness Of Gender Stereotypes ​About Mathematics And Reading: When Girls Improve ​Their Reputation In Math | Delphine Martinot, Céline Bagès, Michel Désert | 2012 |
| 69 | The Costs Of Accepting Gender Differences: The Role ​Of Stereotype Endorsement In Women’s Experience ​In The Math Domain | Toni Schmader, Michael Johns, Marchelle Barquissau | 2004 |
| 70 | Implicit Social Cognitions Predict Sex ​Differences In Math Engagement And ​Achievement | Brian A. Nosek ​University of Virginia ​Frederick L. Smyth | 2011 |
| 71 | Problems In The Pipeline: Stereotype Threat And Women's ​Achievement In High-Level Math Courses | Catherine Good, Joshua Aronson, Jayne Ann Harder | 2008 |
| 72 | Stereotype Threat As Validity Threat: ​The Anxiety–Sex–Threat Interaction | Ana R. Delgado, Gerardo Prieto | 2008 |
| 73 | Separating Implicit Gender Stereotypes Regarding Math ​And Language: Implicit Ability Stereotypes Are Self-Serving ​For Boys And Men, But Not For Girls And Women | Melanie C. Steffens, Petra Jelenec | 2011 |
| 74 | Stereotype Internalization And Women’s Math Performance: ​​The Role Of Interference In Working Memory | Virginie Bonnot, Jean-Claude Croizet | 2007 |
| 75 | Women Are Bad At Math, But I’m Not, Am I?’ Fragile Mathematical Self-Concept ​Predicts Vulnerability To A Stereotype Threat Effect On Mathematical Performance | Friederike X. R. Gerstenberg, Roland Imhoff, Manfred Schmitt | 2012 |
| 76 | Self-Affirmation In Occupational Training: Effects ​On The Math Performance Of French Women Nurses ​Under Stereotype Threat | Anne Taillandier-Schmitt, Catherine Esnard, René Mokounkolo | 2012 |
| 77 | Stereotype Threat And Women’s Math Performance | Steven J. Spencer, Claude M. Steele, Diane M. Quinn | 1999 |
| 78 | Blatant Stereotype Threat And Women’s Math Performance: ​Self-Handicapping As A Strategic Means To Cope With ​Obtrusive Negative Performance Expectations | Johannes Keller | 2002 |
| 79 | The Interplay Among Stereotypes, Performance-Avoidance ​Goals, And Women’s Math Performance Expectations | Jessi L. Smith | 2006 |
| 80 | Stereotype Internalization, ​Math Perceptions, And Occupational ​Choices Of Women ​With Counter-Stereotypical ​​University Majors | Virginie Bonnot, Jean-Claude Croizet | 2007 |
| 81 | Math–Gender Stereotypes In Elementary School Children | Dario Cvencek, Andrew N. Meltzoff, Anthony G. Greenwald | 2011 |
| 82 | Stereotype Threat Reduces Motivation ​To Improve: Effects Of Stereotype Threat ​And Feedback On Women’s Intentions ​To Improve Mathematical Ability | Vincent J. Fogliati, Kay Bussey | 2013 |
| 83 | The Role Of Parents And Teachers In The Development ​Of Gender-Related Math Attitudes | Elizabeth A. Gunderson, Gerardo Ramirez, Susan C. Levine, Sian L. Beilock | 2012 |
| 84 | The Effect Of Negative Performance Stereotypes On Learning | Robert J. Rydell, Michael T. Rydell, Kathryn L. Boucher | 2010 |
| 85 | New Directions For Research On The Role Of Parents ​And Teachers In The Development Of Gender-Related Math ​Attitudes: Response To Commentaries | Elizabeth A. Gunderson, Gerardo Ramirez, Susan C. Levine, Sian L. Beilock | 2012 |
| 86 | Do Parents’ Academic Gender Stereotypes Influence ​Whether They Intrude On Their Children’s Homework? | Ruchi Bhanot, Jasna Jovanovic | 2005 |
| 87 | Forewarning And Forearming ​Stereotype-Threatened Students | Matthew S. McGlone, Joshua Aronson | 2001 |
| 88 | A Beautiful Myth? The Gendering Of Being/Doing ​‘Good At Maths’ | Heather Mendick | 2005 |
| 89 | Gender And Mathematics: Recent Development ​From A Swedish Perspective | Gerd Brandell, Gilah Leder, Peter Nystro¨m | 2007 |
| 90 | Images Of Mathematicians: A New Perspective On The Shortage ​Of Women In Mathematical Careers | Katrina Piatek-Jimenez | 2008 |
| 91 | Shaping Stereotypical Behaviour Through The ​Discussion Of Social Stereotypes | Laura G. E. Smith, Tom Postmes | 2011 |
| 92 | Psychological Processes Underlying Stereotype ​Threat And Standardized Math Test Performance | Katherine E. Ryan, Allison M. Ryan | 2005 |
| 93 | Identity Bifurcation In Response To Stereotype Threat: ​Women And Mathematics | Emily Pronin, Claude M. Steele, Lee Ross | 2004 |
| 94 | The Role Of Performance–Avoidance Goals And Worry In Mediating ​The Relationship Between Stereotype Threat And Performance | Amanda B. Brodish, Patricia G. Devine | 2009 |
| 95 | Gender, Stereotype Threat, And Anxiety: ​​Psychophysiological And Cognitive Evidence | Jason W. Osborne | 2006 |
| 96 | Social Identity Versus Reference Frame Comparisons: ​The Moderating Role Of Stereotype Endorsement | Hart Blanton, Charlene Christie, Maureen Dye | 2002 |
| 97 | Lazy, Dumb, Or Industrious: When Stereotypes ​Convey Attribution Information In The Classroom | Christine Reyna | 2000 |
| 98 | An Examination Of Stereotype Threat Effects On Girls' Mathematics ​Performance | Colleen M. Ganley, Leigh A. Mingle, Allison M. Ryan  Katherine Ryan, Marina Vasilyeva, Michelle Perry | 2013 |
| 99 | The Stereotyped Task Engagement Process: The Role ​Of Interest And Achievement Motivation | Jessi L. Smith, Carol Sansone, Paul H. White | 2007 |
| 100 | Consuming Images: How Television Commercials ​That Elicit Stereotype Threat Can Restrain ​Women Academically And Professionally | Paul G. Davies. Steven J. Spencer, Diane M. Quinn, ​ Rebecca Gerhardstein | 2002 |
| 101 | Making Gender Matter: The Role Of Gender-Based Expectancies ​​And Gender Identification On Women’s And Men’s Math Performance ​​In Sweden | Kimmo Eriksson, Torun Lindholm | 2007 |
| 102 | Confronting Math Stereotypes In The Classroom: Its Effect ​On Female College Students’ Sexism And Perceptions ​Of Confronters | Guy A. Boysen | 2013 |
| 103 | Can Stereotype Threat Explain The Gender Gap In Mathematics ​Performance And Achievement? | Gijsbert Stoet, David C. Geary | 2012 |
| 104 | The Interference Of Stereotype Threat ​With Women’s Generation Of Mathematical ​Problem-Solving Strategies | Diane M. Quinn, Steven J. Spencer | 2001 |
| 105 | A Q-Methodological Study Of Women’s Subjective ​Perspectives On Mathematics | Debra L. Oswald, Richard D. Harvey | 2003 |
| 106 | A Particular Resiliency To Threatening Environments | Michael Inzlicht, Joshua Aronson, Catherine Good, Linda McKay | 2006 |
| 107 | A THREATENING INTELLECTUAL ENVIRONMENT: ​Why Females Are Susceptible To Experiencing Problem-Solving ​Deficits In The Presence Of Males | Michael Inzlicht, Talia Ben-Zeev | 2000 |
| 108 | Effects Of Role Models From Films On Short-Term ​​Ratings Of Intent, Interest, And Self-Assessment Of ​​Ability By High School Youth: A Study Of ​​Gender-Stereotyped Academic Subjects ' | Albert Ziegler, Heidrun Stoeger | 2008 |
| 109 | Do High-Achieving Female Students Underperform In Private? The ​Implications Of Threatening Environments On Intellectual Processing | Michael Inzlicht, Talia Ben-Zeev | 2003 |
| 110 | Preschool Children’s Beliefs About Gender Differences ​In Academic Skills | M. Francisca del Río, Katherine Strasser | 2013 |
| 111 | STEREOTYPE SUSCEPTIBILITY IN CHILDREN: ​Effects Of Identity Activation On Quantitative Performance | Nalini Ambady, Margaret Shih, Amy Kim, Todd L. Pittinsky | 2001 |
| 112 | Stereotype Threat And Gender Differences In ​Performance On A Novel Visuospatial Task | Susan Miller Campbell, Marcia L. Collaer ​ | 2009 |
| 113 | The Gendered Nature Of Competence: Specific And ​​General Aspects Of Self-Knowledge In Social Contexts’ | Laurel J. Bornholt | 2000 |
| 114 | Latina And European American Girls’ Experiences ​With Academic Sexism And Their Self-Concepts ​In Mathematics And Science During Adolescence | Christia Spears Brown, Campbell Leaper | 2010 |
| 115 | Stereotype Threat And Group Differences In Test Performance: A Question ​Of Measurement Invariance | Jelte M. Wicherts, Conor V. Dolan, David J. Hessen | 2005 |
| 116 | Development Of Children’s Math Attitudes: Gender Differences, Key ​​Socializers, And Intervention Approaches | Susan C. Levine, Nancy Pantoja | 2021 |
| 117 | Does Stereotype Threat Influence Performance Of Girls In ​Stereotyped Domains? A Meta-Analysis☆ | Paulette C. Flore, Jelte M. Wicherts | 2015 |
| 118 | Effects Of Gender Stereotypes And Stereotype Threat ​​On Children’s Performance On A Spatial Task | Christine K. Shenouda, ​​Judith H. Danovitch | 2014 |
| 119 | Gender Role Orientation Moderates ​Effects Of Stereotype Activation  On Test Performances | Tobias Tempel, Roland Neumann | 2015 |
| 120 | Gender Stereotypes About Math Anxiety: Ability And Emotional Components | M. Jos´e Justicia-Galiano, M. Eva Martín-Puga, Rocío Linares, Santiago Pelegrina | 2023 |
| 121 | Gender Stereotypes Can Explain The ​Gender-Equality Paradox | Thomas Bredaa, Elyès Jouinia, Clotilde Nappc, Georgia Thebaulta | 2020 |
| 122 | Gender Stereotypes Embedded In Natural Language Are ​​Stronger In More Economically Developed And ​​Individualistic Countries | Clotilde Napp | 2023 |
| 123 | Gender Stereotypes: Implicit Threat To Performance ​​Or Boost For Motivational Aspects In Primary School? | Johanna Maria Hermann, Regina Vollmeyer | 2022 |
| 124 | Gendered Beliefs About Mathematics ​​Ability Transmit Across Generations Through ​​Children’s Peers | Alex Eble , Feng Hu | 2022 |
| 125 | Implicit Self-Stereotyping Under Eye Gaze: The Effects Of ​​Gaze Cues On Implicit Math Identity Among Women | Yusuke Karouji, Takashi Kusumi | 2015 |
| 126 | Implicit Gender Stereotypes And Essentialist Beliefs Predict Preservice Teachers’ Tracking Recommendations | Miriam Nürnberger, Josef Nerb, Florian Schmitz, Johannes Keller, Stefan ​Sütterlin | 2016 |
| 127 | Implicit Math-Gender Stereotype Present In Adults But Not In 8th ​Grade | Kyle Morrissey, Darcy Hallett, Aishah Bakhtiar, Cheryll Fitzpatrick | 2019 |
| 128 | Interaction Of Task Difficulty And Gender Stereotype Threat ​With A Spatial Orientation Task In A Virtual Nested Environment | Craig Allison, Edward S. Redhead, Wai Chan | 2017 |
| 129 | Is Emma Or Liam The Top Scorer In Math? The Effects ​​Of A Counter‑Stereotypical Role Model On Math Achievement | Nadia Leroy, Sylvain Max, Pascal Pansu | 2022 |
| 130 | Leaderboards In A Virtual Classroom: A Test Of Stereotype ​Threat And Social Comparison Explanations For Women's ​Math Performance | Katheryn R. Christy, Jesse Fox | 2014 |
| 131 | Math Question Type And Stereotype Threat: ​ Evidence From Educational Settings | Lucy C. Davies, Mark Conner, Constantine Sedikides , Russell R. C | 2016 |
| 132 | Math–Gender Stereotypes And Math-Related Beliefs In Childhood And ​Early Adolescence | Maria Chiara Passolunghi, Tania Irene Rueda Ferreira, Carlo Tomasetto | 2014 |
| 133 | Mental Rotation And Mathematics: Gender-Stereotyped Beliefs And ​Relationships In Primary School Children | Angelica Moè | 2018 |
| 134 | Not The Sum Of Its Parts: Decomposing Implicit Academic ​Stereotypes To Understand Sense Of Fit In Math And English | Patricia N. Gilbert, Laurie T. O’Brien, Donna M. Garcia, David M. Marx | 2015 |
| 135 | Numbers For Boys And Words For Girls? Academic Gender Stereotypes ​​Among Chinese Parents | Jing Li, Eman Faisal, Ahmed Al Hariri | 2022 |
| 136 | Parents’ Math Gender Stereotypes And Their Correlates: ​​An Examination Of The Similarities And Differences Over The Past 25 ​​Years | Christine R. Starr, Yannan Gao, Glona Lee, Nayssan Safavian, Charlott Rubach, Anna‑Lena Dicke, Jacquelynne S. Ecclesi Sandra D. Simpkins | 2022 |
| 137 | Rethinking Employment Discrimination Harms | Jessica L. Roberts | 2016 |
| 138 | Self-Concept Explains Gender ​​Differences In Mental Rotation ​Performance After Stereotype ​​Activation | Martina Rahe, Linda Schürmann, Petra Jansen | 2023 |
| 139 | Teacher Gender, Student Gender, And Primary School ​Achievement: Evidence From Ten Francophone African ​Countries | Jieun Lee, Dong-Eun Rhee, Robert Rudolf | 2019 |
| 140 | The Effects Of Gender Composition On Women’s Experience In Math Work Groups | Sarah S. Grover, Tiffany A. Ito, Bernadette Park | 2017 |
| 141 | The Gender Gap In STEM Fields: The Impact Of The Gender Stereotype Of Math And Science On Secondary Students’ Career Aspirations | Elena Makarova, Belinda Aeschlimann, Walter Herzog | 2019 |
| 142 | The Impact Of Math-Gender Stereotypes On Students’ Academic ​Performance: Evidence From China | Yilei Luo, Xinqi Chen | 2024 |
| 143 | The Roots Of Stereotype Threat: When Automatic Associations Disrupt Girls’ ​Math Performance | Silvia Galdi, Mara Cadinu, Carlo Tomasetto | 2014 |
| 144 | The Effect Of Gender Stereotypes On Young ​Girls’ Intuitive Number Sense | Antonya Marie GonzalezI, Darko Odic, Toni Schmader, Katharina Block, Andrew ​Scott Baron | 2021 |
| 145 | The Effect Of Mindfulness And Stereotype Threat In Mental Rotation: A Pupillometry Study | Robert Bauer, Leonardo Jost, Petra Jansen | 2021 |
| 146 | The Interest Gap: How Gender Stereotype Endorsement ​​About Abilities Predicts Differences In Academic Interests | Isabelle Plante, Paul A. O’Keefe, Joshua Aronson, ​​Catherine Fréchette‑Simard, Mélissa Goulet | 2019 |
| 147 | The Negative Effects Of Stereotype ​​Threat On Women’s Spatial Ability: ​​The Moderating Role Of Resilience | Zhen Wang, Li Zhao, Yiwen Shan, Jian Guan | 2024 |
| 148 | The Psychosocial Experience Of High School Girls Highly ​Susceptible To Stereotype Threat: A Phenomenological Study | Katherine Picho | 2016 |
| 149 | The Role Of Implicit Gender Spatial Stereotyping In Mental Rotation ​Performance | Francesca Guizzoa, Angelica Moèb, Mara Cadinua, Chiara Bertollia | 2019 |
| 150 | When Do Gender Stereotypes Impair Math Performance? ​A Study Of Stereotype Threat Among Ugandan Adolescents | Katherine Picho, Toni Schmader | 2018 |
| 151 | Our Future Scientists: A Review Of Stereotype Threat ​​In Girls From Early Elementary School To Middle School | Isabelle Régner, Jennifer R. Steele, ​​Nalini Ambady, ​​Catherine Thinus-Blanc, ​​Pascal Huguet | 2014 |
| 152 | Revealing Stereotype Threat Effects And Women’s Maths Performance The Moderating Role Of Mathematical Anxiety | Daniel Pérez-Garín, Antonio Bustillos, Fernando Molero | 2017 |
