# Supplementary material for: Unpacking mathematical gender stereotypes: trends and directions from 25 years of research
Source: Front Psychol. 2025 Nov 18;16:1660583. doi: 10.3389/fpsyg.2025.1660583 (PMC12669155; doi:10.3389/fpsyg.2025.1660583)
Supplement: Supplementary file 3 [file Supplementary_file_3.docx]

**PRISMA Items Not Implemented and Their Reasons**

|  |  | PRISMA Item | Reason/Explanation |
| --- | --- | --- | --- |
| Effect measures | 12 | Specify for each outcome the effect measure(s) (e.g. risk ratio, mean difference) used in the synthesis or presentation of results. | No specific effect measures such as risk ratios or mean differences were used in this systematic review, as the study focused on qualitative content analysis of mathematical gender stereotypes rather than quantitative synthesis of outcomes. |
| Synthesis methods | 13b | Describe any methods required to prepare the data for presentation or synthesis, such as handling of missing summary statistics, or data conversions. | No data conversions or statistical imputations were performed, as the study used a qualitative content analysis approach. Missing or unclear data were recorded as “not reported” and excluded from category-specific analyses. |
| Results of individual studies | 19 | For all outcomes, present, for each study: (a) summary statistics for each group (where appropriate) and (b) an effect estimate and its precision (e.g. confidence/credible interval), ideally using structured tables or plots. | As this study employed qualitative content analysis and did not perform meta-analysis, summary statistics, effect estimates, and their precision (e.g., confidence intervals) are not applicable and thus not presented. |
| Results of syntheses | 20b | Present results of all statistical syntheses conducted. If meta-analysis was done, present for each the summary estimate and its precision (e.g. confidence/credible interval) and measures of statistical heterogeneity. If comparing groups, describe the direction of the effect. | No statistical syntheses, including meta-analyses, were conducted in this study due to the nature of the qualitative content analysis approach. |
| Results of syntheses | 20c | Present results of all investigations of possible causes of heterogeneity among study results. | No formal investigations into causes of heterogeneity were conducted, as this study is based on qualitative content analysis. |
| Results of syntheses | 20d | Present results of all sensitivity analyses conducted to assess the robustness of the synthesized results. | No sensitivity analyses were performed because the study employed qualitative content analysis, which does not involve statistical synthesis requiring such assessments |
| Reporting biases | 21 | Present assessments of risk of bias due to missing results (arising from reporting biases) for each synthesis assessed. | No formal assessment of risk of bias due to missing results (reporting bias) was conducted, as this study employed qualitative content analysis and included all available data from the selected studies. |
| Certainty of evidence | 22 | Present assessments of certainty (or confidence) in the body of evidence for each outcome assessed. | No formal assessment of certainty or confidence in the evidence was performed because the study used qualitative content analysis rather than quantitative synthesis |
| Registration and protocol | 24b | Indicate where the review protocol can be accessed, or state that a protocol was not prepared. | This systematic review was not registered and no protocol was prepared prior to conducting the study |
| Registration and protocol | 24c | Describe and explain any amendments to information provided at registration or in the protocol. | No protocol was prepared or registered; therefore, no amendments were made |
