# Supplementary material for: Unpacking mathematical gender stereotypes: trends and directions from 25 years of research
Source: Front Psychol. 2025 Nov 18;16:1660583. doi: 10.3389/fpsyg.2025.1660583 (PMC12669155; doi:10.3389/fpsyg.2025.1660583)
Supplement: Supplementary file 4 [file Supplementary_file_4.docx]

The coding process, based on these categories, was carried out as follows:

**Field**

This category was analyzed by considering the subject area in which the article was conducted, applied, or related to. For example, if a study aimed to "examine communicative strategies to help female students cope with stereotype threat," the field was coded as communication and education/educational research. In another study, if the aim was to "test the effects of a stereotype threat condition (vs. control group) among middle school girls in standard and honors math classes and examine gender identity as a moderator," the field was coded as women's studies and psychology. Additionally, this classification was based on the "Research Areas" section provided by Web of Science.

**Subject Matters**

In the analysis of this category, the subject matter section of the code and category list developed by Baş and Özturan Sağırlı (2017) [56] was reorganized to align with the concept of mathematical gender stereotypes. During this process, the problem, sub-problem, and objective sections of the mathematical gender stereotypes-themed articles included in the study were read several times to define the codes and categories. The subjects of the studies were initially assigned codes such as gender, achievement, anxiety, language ability stereotypes, and mental rotation performance. However, it was later realized that these categories, which were initially defined as drafts, were explored for different research purposes. As a result, it was decided that the relationships among these topics should be clearly specified. For instance, some emerging themes included testing the moderating role of mathematics anxiety in the relationship between stereotype threat and impaired performance, as well as investigating whether the endorsement of mathematical gender stereotypes varies by gender. The final categorization consists of five distinct categories. Additionally, only problem statements or research objectives related to mathematical gender stereotypes were considered for evaluation. For example,

1- Female students will achieve lower math test scores than male students overall.

2- Forewarning female students about stereotype threat and advising them to suppress stereotype-related thoughts will impair their math test scores (relative to a no-forewarning control condition).

3- Forewarning female students about stereotype threat and reminding them of a self-relevant stereotype with positive performance expectations will improve their math test scores (relative to the control condition).

Only the second and third sub-problems from the study, as mentioned above, were analyzed within the 'subject matter' category, specifically in relation to stereotype threat.

**Method**

In terms of research methods, each paper was categorized based on a predefined code and category list. In some of the examined studies, the methodology was not explicitly specified. In such cases, the researcher assigned an appropriate code by considering the subject matter, data, sample, data tools, and analysis process.

**Sample**

The samples utilized in the research were categorized into eleven groups: early childhood, primary school, middle school, high school, undergraduate, graduate, teachers, school principals, parents, adults, and documents.

**Data Collection Tools**

In the analysis of this category, tools directly related to the term "mathematical gender stereotypes" were considered. For example, a study aimed at investigating whether the internalization of the stereotype of mathematical inferiority directed at women affects their math performance. The tool used to assess math performance was not included in the analysis. However, the instrument used to measure endorsement of mathematical gender stereotypes was examined within the category of open-ended questionnaires/scales/tests.

For each instrument, instrument family/name, reliability (Cronbach’s α; test–retest/split-half), structural validity (CFA fit: CFI/TLI/RMSEA) were coded.

**Data Analysis**

This section focuses solely on the data analysis methods utilized for examining mathematical gender stereotype-related data.

**Country**

In this category, the country to which the study sample belongs was analyzed.

**Year**

This category analyzes the publication years of the articles reviewed.

**Definitions**

Within the definitional analysis, we systematically examined the introduction, theoretical framework, and/or literature review sections of the included articles. The unit of analysis comprised sentences and paragraphs in which the studies constructed the concept of “stereotype” (i.e., offered a definition or conceptualization). Findings, measurement instruments, and results/discussion sections were deliberately excluded from this stage.

The analysis followed a descriptive–interpretive thematic coding approach. In a first cycle, texts were read closely and explicit definitions, operational statements, and conceptual framings of stereotypes were open coded. In a second cycle, these initial codes were clustered into a hierarchical scheme of definitional axes and their subthemes. Code labels were retained in English to align with terminology used in the field.

Each definitional statement was assigned to the most appropriate parent axis and subtheme. For example, when reviewing stereotype threat definitions, authors frequently emphasized the “risk of confirming the stereotypes,” and statements such as “Stereotype threat can be defined as the risk of confirming an existing negative stereotype of the own group.” were coded under Stereotype Threat → Risk of Confirming the Stereotypes. Similarly, definitions of mathematical gender stereotypes that foregrounded male superiority were grouped under Mathematical Gender Stereotypes → Superiority of Males in Math, with an additional subcode “Males Are Better/Competent/Talented in Math.” For instance, “The view that men are better in mathematics is widely held and these stereotypes could affect females’ mathematical performance (Ertl et al., 2017; Luttenberger et al., 2018).” was coded within this subcategory.

Where articles articulated multiple conceptual definitions, each was coded independently and multiple coding was permitted. Throughout coding, definitions, inclusion/exclusion rules, and representative excerpts were documented and iteratively refined in a codebook; themes were stabilized upon reaching saturation. Finally, the relative prevalence of each axis and subtheme was summarized using frequency counts, and the hierarchical structure was presented schematically.

**Conclusions**

We conducted a targeted content analysis to identify the conclusions reported in studies on gender stereotypes in mathematics. Specifically, we systematically examined the abstract, results/findings, and conclusions sections of the included articles. The unit of analysis comprised sentences and paragraphs that articulated empirical outcomes or principal inferences. The introduction, theoretical framework, and literature review sections were deliberately excluded from this phase.

The analysis followed a descriptive–interpretive thematic coding approach. In the first cycle, the texts were closely examined and the reported conclusions of the studies were open coded. In the second cycle, these initial codes were clustered within a hierarchical framework encompassing overarching themes and their corresponding subthemes.

Findings were examined under six categories: qualitative, descriptive, correlational, mediational, experimental, and meta-analytic. Under the qualitative heading, within the School Ecology and Representation code, we introduced a subcode for teachers’ biased practices and beliefs, documenting classroom discourse and expectation patterns that varied by gender. Specifically, boys were more often addressed with language implying expectation and directive intent, whereas discourse toward girls was predominantly encouraging and volitional. An illustrative classroom excerpt is:

“(To a boy, lesson 3): You understand the work (scowling at him). I believe in you. So, get up and go to the blackboard and solve the exercise.
In contrast, girls were encouraged, rather than expected, to succeed, for example:
(To a girl, lesson 6): Would you like to go to the blackboard and try to solve the problem?”

Within the descriptive statistics findings, a code for domain/identity stereotypes was introduced. Under this code, a subcode for mathematical gender stereotypes was developed, which further included an additional subcode capturing the age at which beliefs in mathematical gender stereotypes first appear. For instance, the finding that “… Results showed that 3rd grade boys and girls systematically rated their gender ingroup as better at math at the explicit level.” was categorized within this framework, illustrating the early emergence of explicit ingroup-based stereotypical beliefs about mathematical competence.

In the correlational corpus, constructs were organized under gender stereotype endorsement, mathematical gender stereotype beliefs, counter-stereotypical role model, and related categories, and systematically mapped to examined predictors and outcomes. A representative study tested the association between exposure to a counter-stereotypical role model (a female top math scorer in class) and mathematics performance using multilevel analyses of 1,043 sixth-graders nested in 46 classes. Performance declined over the school year for all students; however, being female exerted a buffering effect on this decline. The study supported the cross-level interaction hypothesis (H2), indicating that the greatest benefits accrued to girls in classes where the top math scorer was a girl. Collectively, these results suggest that counter-stereotypical female exemplars mitigate stereotype-related performance erosion via interactional (context × identity) mechanisms rather than simple gender main effects.

Mediational models were coded under the headings independent variable (IV), mediator/moderator (M), dependent variable (DV), and direction of effect. An illustrative coding is as follows:

IV: Stereotype activation (activated gender stereotypes)
M (moderator): Gender role orientation (feminine orientation derived from the Bem Sex Role Inventory; continuous)
DVs: Mental rotation performance; Mathematics performance
Direction of effect: Among female participants, higher feminine gender-role orientation amplified the negative association of stereotype activation with both mental rotation and mathematics performance.

This pattern indicates a conditional (moderation) effect rather than a simple main effect of gender, suggesting that the detrimental impact of stereotype activation on performance strengthens as feminine gender-role orientation increases.

Experimental studies were synthesized within a systematic coding framework under three headings: context, domain effect, and direction of effect. In this approach, we coded (i) the nature of the experimental induction (e.g., threat, counter-stereotypical message, “girls/boys are better” instructions), (ii) the targeted cognitive/affective domain (e.g., mental rotation, mathematics achievement), and (iii) the outcome direction by gender, each as separate dimensions.

As an illustration, a study that inserted gender-difference instructions between two mental-rotation tests (“boys better,” “girls better,” “no gender difference”), examined the time × gender interaction, and reported female-advantaging/male-disadvantaging effects was coded as follows:
Context: Stereotype lift
Domain Effect: Mental rotation performance
Direction of Effect: Female (+), Male (–)

This coding scheme explicitly delineates for whom, in which domain, and in what direction experimental contexts exert effects, thereby providing a comparable, integrative summary of how threat/lift manipulations operate through context × identity × domain interactions.

Meta-analytic studies were planned to be synthesized by context and domain effect. In the corpus, eligible meta-analyses were identified only under the stereotype threat context and the mathematics achievement domain (broadened here to MSSS: mathematics, science, and spatial skills), and their effect sizes were coded directly. For example: A meta-analysis examining the impact of stereotype threat on girls’ performance in mathematics, science, and spatial skills (MSSS) tests. Random-effects and mixed-effects models across k = 47 effect sizes.Pooled Estimate: d = −0.22, significantly different from zero. This study was codded as effects of stereotype threat on mathematics performance d = −0.22 (significant).
